# Heteroatom-Doped Molybdenum Disulfide Nanomaterials for Gas Sensors, Alkali Metal-Ion Batteries and Supercapacitors

**DOI:** 10.3390/nano13152182

**Published:** 2023-07-26

**Authors:** Lyubov G. Bulusheva, Galina I. Semushkina, Anastasiya D. Fedorenko

**Affiliations:** Nikolaev Institute of Inorganic Chemistry SB RAS, 3 Acad. Lavrentiev Ave., 630090 Novosibirsk, Russia; spectroscopy@mail.ru (G.I.S.); fedorenko@niic.nsc.ru (A.D.F.)

**Keywords:** MoS_2_, substitutional doping, gas sensors, electrochemical capacitors, rechargeable batteries

## Abstract

Molybdenum disulfide (MoS_2_) is the second two-dimensional material after graphene that received a lot of attention from the research community. Strong S–Mo–S bonds make the sandwich-like layer mechanically and chemically stable, while the abundance of precursors and several developed synthesis methods allow obtaining various MoS_2_ architectures, including those in combinations with a carbon component. Doping of MoS_2_ with heteroatom substituents can occur by replacing Mo and S with other cations and anions. This creates active sites on the basal plane, which is important for the adsorption of reactive species. Adsorption is a key step in the gas detection and electrochemical energy storage processes discussed in this review. The literature data were analyzed in the light of the influence of a substitutional heteroatom on the interaction of MoS_2_ with gas molecules and electrolyte ions. Theory predicts that the binding energy of molecules to a MoS_2_ surface increases in the presence of heteroatoms, and experiments showed that such surfaces are more sensitive to certain gases. The best electrochemical performance of MoS_2_-based nanomaterials is usually achieved by including foreign metals. Heteroatoms improve the electrical conductivity of MoS_2_, which is a semiconductor in a thermodynamically stable hexagonal form, increase the distance between layers, and cause lattice deformation and electronic density redistribution. An analysis of literature data showed that co-doping with various elements is most attractive for improving the performance of MoS_2_ in sensor and electrochemical applications. This is the first comprehensive review on the influence of foreign elements inserted into MoS_2_ lattice on the performance of a nanomaterial in chemiresistive gas sensors, lithium-, sodium-, and potassium-ion batteries, and supercapacitors. The collected data can serve as a guide to determine which elements and combinations of elements can be used to obtain a MoS_2_-based nanomaterial with the properties required for a particular application.

## 1. Introduction

Molybdenum disulfide (MoS_2_) is a layered compound, where the layers are three atoms thick due to the sandwich-like S–Mo–S structure [1]. Within the layer, molybdenum forms strong bonds with six sulfur atoms, while sulfur atoms from neighboring layers interact through weak van der Waals (vdW) forces. Depending on the coordination of sulfur atoms to the molybdenum atom and the vertical stacking of layers, MoS_2_ can form several crystal phases [2]. The hexagonal 2H phase is the most thermodynamically stable and constitutes about 80% of the natural mineral molybdenite [3]. Owing to the easy sliding of layers under applied shear, MoS_2_ finds industrial use as a dry lubricant [4]. Another practical application of MoS_2_ is oil hydrodesulfurization [5]. The catalytic properties of MoS_2_ are associated with chemically active edge states. The large interlayer distance of 0.62 nm makes MoS_2_ attractive for intercalation reactions [6]. Such reactions proceed most easily with lithium-containing compounds. The electrochemical intercalation of lithium ions into MoS_2_ was studied at the end of the last century [7,8] in the search for a suitable cathode material for rechargeable lithium-ion batteries [9]. However, the lithiation voltage of 1.1–2.0 V for MoS_2_ [10] is too low to provide a high energy density of a full cell.

Interestingly, the delamination of 2H-MoS_2_ crystals to monolayers by the peeling technique was carried out in 1966 [11], forty years before graphene was obtained from graphite in a similar way. After it was found that graphene has unique properties as compare to the bulk parent material [12], interest in layered materials increased significantly [13], and MoS_2_ has become the second most intensively studied material from this family [14]. In contrast to graphene, MoS_2_ is a semiconductor with a varied band gap depending on the number of adjacent layers [15]. This property makes MoS_2_ a candidate for use in electronic and optoelectronic circuits, memory elements, etc. [16,17]. The sulfur-rich surface is capable of adsorbing various substances; therefore, MoS_2_ is promising for water purification [18,19] and detection of chemicals in liquid and gaseous environments [20,21]. Sulfur has a strong affinity for heavy metals, and depending on the nature of the metal, adsorption occurs through complexation, electrostatic interaction, or redox reaction mechanisms, which were discussed in the critical review [19]. Detailed data on the development of MoS_2_ nanostructures for real-time detection of biomarkers, drugs, and food ingredients were given in the review [22]. Electrochemical MoS_2_-based sensing elements can be used in portable and wearable devices for monitoring blood glucose levels, on-site detection of inorganic ions, and undesirable additives in water and food [22], as well as for recording human motion, since they show a high response over a wide operation pressure range [23]. The appropriate band gap and significant change in conductivity due to charge transfer from/to adsorbed molecules make MoS_2_ useful for gas detection. Reviews in this field of application emphasize the need to find suitable modifiers for MoS_2_ to achieve ultra-high selectivity in gas sensing [24,25]. Energy applications of MoS_2_ can be classified as energy storage and energy generation [26] The first is provided by batteries and electrochemical capacitors (or supercapacitors), the most studied process of generating energy using MoS_2_ as a catalyst is the hydrogen evolution reaction (HER) [27,28].

Most applications of MoS_2_ require tuning the band gap and/or creating the necessary active sites. These characteristics can be purposefully changed by replacing Mo and S with other chemical elements. It was shown that doping with heteroatoms improves the tribological properties of MoS_2_ [29], enhances the efficiency of water purification [30], electrocatalytic water splitting [31], and detection of NO_2_ gas [32]. Ni and Co located at the edges of MoS_2_ clusters were revealed to promote the desulfurization of organic compounds [33]. Since 2018, more than two thousand papers in the field of MoS_2_-based nanomaterials have been published annually, and so, the description and critical analysis of the current state of research is constantly required. Strategies for inserting transition metals into MoS_2_ nanomaterials were described in [34]. The review considered the change in the electrical conductivity of MoS_2_ upon substitutional doping and catalytic properties for HER, hydrodesulfurization reactions, and reduction of carbon dioxide to methanol. An overview of developments on substitutional doping of layered transition metal dichalcogenides, including MoS_2_, is presented in [35]. It was concluded that many foreign elements could be incorporated in the lattice of these materials, which opens up wide opportunities for the engineering of electronic structure. Changes in the structure, electrical, optical, and magnetic properties of MoS_2_ crystals due to various single atom dopants were discussed in [36]. However, there is still no comprehensive review considering the effect of heteroatoms on the properties of MoS_2_ nanomaterials for gas sensors and electrochemical energy storage devices, the efficiency of which strongly depends on the conductivity of the nanomaterial and its interaction with the adsorbate. 

This gap is filled by the present review paper devoted to the performance of heteroatom-doped MoS_2_ nanomaterials in chemiresistive gas sensors, alkali metal-ion batteries, and supercapacitors. Modern research cannot be imagined without theoretical calculations evaluating the thermodynamic possibility of the formation of a particular structure, changes in the band gap of a semiconductor during doping, the binding energy with an adsorbate, and the charge transfer accompanying this process. Therefore, we first analyzed theoretical studies on the change in the electronic structure of MoS_2_, as a result of substitutional doping in Section 2.1. Section 2.2 describes the experimental methods, which allow identifying the incorporation of heteroatoms into the MoS_2_ lattice. Section 3 is devoted to chemiresistive MoS_2_ sensors, it describes and analyzes the data obtained for undoped MoS_2_, compares them with data for heteroatom-doped MoS_2_, and presents the adsorption energies and charge transfer values in models calculated using density functional theory (DFT). Section 4 provides electrochemical characteristics of heteroatom-doped MoS_2_ nanomaterials in lithium-ion batteries (LIBs), sodium-ion batteries (SIBs), potassium-ion batteries (PIBs), and supercapacitors. The main results of the review and the challenges are given in Section 5. 

## 2. Foreign Elements in MoS_2_

**Figure 1:**
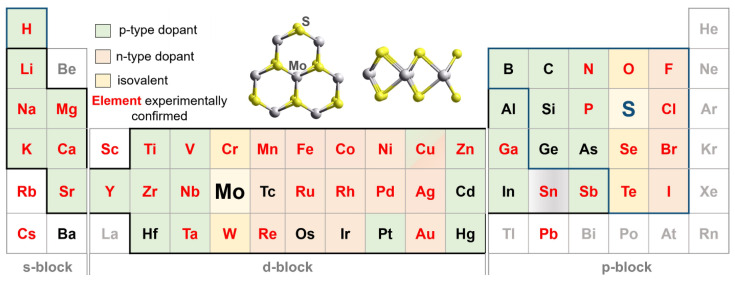
Structures show the MoS_2_ fragment in top and side projections. Elements considered as heteroatoms in MoS_2_ in theoretical works are located in the shaded cells of the periodic table. Elements located in green and cream cells cause p- and n-type doping, respectively. Elements in yellow cells are isovalent to molybdenum or sulfur, which are found in light yellow cells. Gray color of the cell with Sb indicates the metallic type due to doping. Elements of red color were introduced into MoS_2_ experimentally.

Sulfur belongs to the chalcogen family and in compounds with metals has an oxidation state of −2. Since there are two sulfur atoms per molybdenum atom in MoS_2_, molybdenum oxidation state is +4. The incorporation of foreign atoms into the MoS_2_ structure can occur along the cationic and anionic sublattices. The elements considered in theoretical studies as possible substituents for Mo and S are shown in Figure 1 in shaded cells of the periodic table of chemical elements. 

Substitution by an element belonging to the same group is isovalent and basically causes a shift in the energy levels of the resulting compound. The introduction of elements with a different number of valence electrons than those of Mo and S atoms can additionally locally distort the lattice and affect the character of the chemical bonding. In this section, we will first consider these changes revealed by DFT calculations and then briefly describe the experimental data on the ability of MoS_2_ to include foreign elements and the methods for identifying doping.

### 2.1. Theoretical Aspects

DFT calculations are used to estimate the formation energy of alloys and heteroatoms, the thermodynamically preferable configurations of foreign atoms, and the changes in the electronic structure of the compounds that accompany the replacement. Most of the calculations are carried out for monolayers. 

The formation energy of an alloy Mo_1−x_M_x_S_2_ or MoS_2−x_Y_x_-containing metal (M) or nonmetal (Y) heteroatom is defined as:

E_f_ = E_alloy_ − (1 − x)E_MoS2_ − xE_MS2(MoY2)_, (1)

where the terms are the total energies of the optimized alloy structure and the optimized hexagonal constituents MoS_2_ and metal sulfide MS_2_ or molybdenum compound MoY_2_ per formula unit.

The formation energy (formation enthalpy) of a substitutional dopant can be calculated as the difference between the total energies of a monolayer with a heteroatom (E_H_) and an ideal MoS_2_ monolayer, taking into account the energy (or chemical potential) of an individual native atom (E_N_) and an individual inserting atom (E_A_):

E_f_ = E_H_ − E_MoS2_ + E_N_ − E_A_ (2)

Some authors consider the filling of an atomic vacancy present in the MoS_2_ layer. In this case, the formation (or binding) energy is calculated relative to the layer with the vacancy:

E_f_ = E_V_ + E_A_ − E_H_, (3)

where E_V_ is the total energy of optimized supercell with an atomic vacancy. 

For a uniform distribution of a foreign atom in the crystal, the central Mo or S atom in the supercell is replaced by another element and the portion of the dopant depends on the size of the supercell. Since this size and the calculation method used affect the energy of the system, we will only consider the trends obtained in a particular work.

#### 2.1.1. Molybdenum Replacement

In the hexagonal layer, the molybdenum atom has six molybdenum neighbors (top projection in Figure 1) located at a distance of 0.316 nm, and DFT calculations show these atoms weakly interact with each other [37]. The top of the valence band of MoS_2_ is mainly formed by the Mo 4d states, while the low conduction band consists of the Mo 4d and S 2p orbitals [38]. 

Metals from s- and p-blocks. Alkali metals (Li, Na, and K), alkaline earth metals (Mg, Ca, and Sr), and boron group metals (Al, Ca, and In) were considered in [39] to assess the stability of doped MoS_2_ monolayers. The impurity concentration was 6.25%. Calculations showed that the foreign metal–sulfur bond is longer than the Mo–S bond. The greatest energy gain, determined by Equation (3), was obtained when the molybdenum vacancy was filled with Al, and among the considered metals, the filling with K was energetically less favorable. All introduced metals created impurity states near the top of the valence band. The study of p-block metals Ga, Sn, and Sb revealed that they prefer to be located on the edges rather in the MoS_2_ basal plane [40]. Sulfur-rich conditions were shown to be favorable for replacing Mo with Sb [41]. The heteroatom acts as the predominant p-type dopant and its incorporation in an amount of 4% into the MoS_2_ monolayer leads to the appearance of five states inside the band gap. These states are the result of re-hybridization of the levels induced by a single Mo vacancy and the Sb 5s orbital. 

Metals from d-block. Cr and W belong to the same metal group of the periodic table of the periodic table of the elements (Figure 1) and the substitution of Mo by these metals is isovalent. The introduction of isolated Cr atoms into a bulk hexagonal MoS_2_ crystal with a concentration of 6.25 and 12.5% only slightly changes the band gap (by 0.05–0.07 eV), retaining the semiconducting nature of the compound [42]. The lattice mismatch of hexagonal MoS_2_ and CrS_2_ monolayers is ~4%, and the formation of Mo_1−x_Cr_x_S_2_ alloys was studied at x = 0.2, 0.4, 0.6, and 0.8 [43]. The formation of alloys was found to be endothermic, and so, they would prefer to segregate at 0 K. However, very low energies calculated by Equation (1) suggest their formation under ambient conditions. Due to desirable Cr–Cr interactions, these atoms tend to form ordered lines in the MoS_2_ lattice. The strong hybridization of the Cr 3d and Mo 4d orbitals at the edge of the valence band and the dominant contribution of the Cr 3d orbitals to the edge of the conduction band cause a decrease in the band gap of the alloy with increasing concentration of Cr. 

Hexagonal monolayers of MoS_2_ and WS_2_ have close lattice constants (the difference is ~0.2%), and so, the structural deformation due to the substitution of one metal for another is negligible, and the band gap depends on the charge exchange [44]. The charge transfer from weak Mo−S bonds to the nearest strong W−S bonds [45] causes negative formation enthalpies of hexagonal Mo_1−x_W_x_S_2_ for the entire range 0 ≤ x ≤ 1 [46]. At T = 0 K, solid solutions tend to chemical ordering, rather than clustering. However, random solid solutions can stabilize at elevated temperature due to the increasingly strong contribution of configuration entropy. The most stable alloys are formed in the x range from 0.33 to 0.66 [45,46,47,48]. In this range, the alloy formation energy is practically independent of the dopant concentration, and a large degree of disorder can be realized in the course of synthesis [49]. Since there are many energetically degenerate structures for a given composition, the alloys are disordered in the long range, while the position of foreign atoms in the lattice has little effect on the band gap [45]. The order–disorder transition temperatures for the Mo_1−x_W_x_S_2_ monolayers with 0.33 < x < 0.66 are estimated at approximately 88–102 K [50]. The W impurity in MoS_2_ causes a decrease in the band gap to a minimum value at x = 0.33 [47]. With a further increase in the content of tungsten, a parabolic rise in the band gap occurs due to the dominant contribution of W orbitals to the conduction band edge [51]. The band gap of the ordered phase is smaller than the band gap of the disordered phase for each concentration W [52]. The valence band edge only correlates with the x-composition, while the conduction band edge is very sensitive to the degree of disorder.

**Figure 2:**
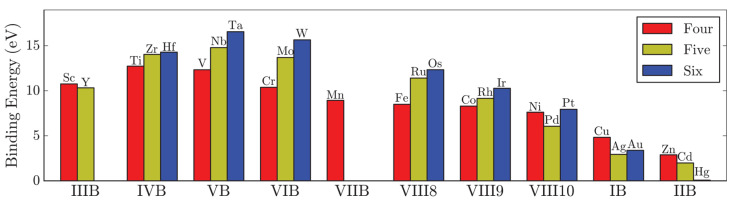
Binding energies of metal atoms from the four, five, and six periods with a molybdenum vacancy in monolayer MoS_2_, taken with permission from [53]; Copyright 2013, American Physical Society.

The screening of 26 metals with a concentration of 6.25% in a MoS_2_ monolayer was performed in [53]. The dopant formation energy was calculated using Equation (3); the results are shown in Figure 2. The higher the energy, the energetically easier it is to fill the molybdenum vacancy with an element. The gain in self-substitution of Mo is 13.5 eV. The most preferred metals for insertion are Ta, W, and Nb, and the introduction of transition metals Hg, Cd, Zn, Ag, Au, and Cu is difficult. 

The introduction of metals into the hexagonal MoS_2_ lattice having fewer and more valence electrons than the molybdenum atom leads to the formation of acceptor levels (p-type doping) and donor levels (n-type doping), respectively.

Six metals, V, Nb, Ta, Mn, Fe, and Co, were used to obtain 4% impurities in MoS_2_ monolayers [54]. The formation energy was calculated using Equation (2). The negative formation energies found for Nb and Ta indicate that these elements are desired for incorporation into the MoS_2_ lattice. For Nb, it was shown that the value became more negative as the number of dopants increased [55]. Among the studied magnetic transition metals, the lowest formation energy was determined for Mn-doped MoS_2_ [54]. The energetically favorable formation of less than 5% Mn defects in MoS_2_ monolayers was also shown in [56]. The thermodynamic driving force of clustering of Mn dopants was predicted in [57]. Replacing Mo with metals having one less valence electron (V, Nb, and Ta) produced the states near the valence band, i.e., induced p-type doping [54]. The transition metals Mn, Fe, and Co have one, two, and three valence electrons in excess with respect to Mo, and these electrons occupied the impurity states appeared in the band gap [58]. The Mn dopant created impurity states close to the edge of the conduction band, while the corresponding states induced by Fe and Co were below. The small separation between the Fermi level and the conduction band of 0.48 eV makes Mn the most attractive for n-type doping.

The heteroatoms Y, Zr, Nb, Re, Rh, Ru, Pd, Ag, Cd were introduced into an MoS_2_ monolayer at a concentration of 4% and the formation energy was calculated according to Equation (2) [59]. It was shown that Nb and Zr are the most preferred substitutional elements. Re has one extra electron as compared to Mo, and its introduction into MoS_2_ creates a donor level 0.2 eV below the conduction band. This level is occupied by rhenium electron with the d_z_^2^ orbital character [60]. Rhenium can form complexes with a sulfur vacancy that quench n-type doping induced by isolated heteroatoms.

**Figure 3:**
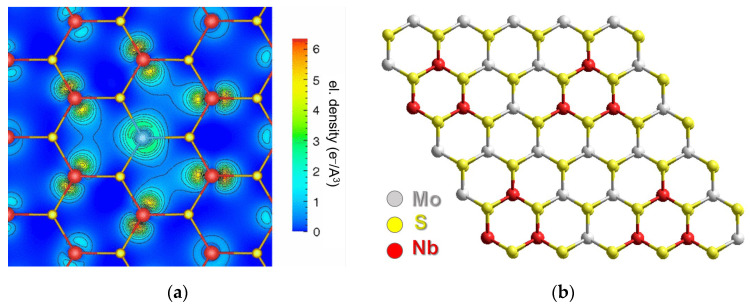
(**a**) Electron density distribution of impurity state associated with the introduction of Nb atom (blue sphere) into MoS_2_; taken with permission from [61]; Copyright 2008, American Physical Society. (**b**) Model of MoS_2_ with 33% Nb concentration as triatomic clusters centered on a hole of a hexagon.

An increase in the occupation of the valence d-orbitals in the Re, Ru, Rh, Pd, Ag, Cd set results in a progressive increase in the states in the band gap [59]. Nb, Zr, and Y have, respectively, one, two, and three electrons less than Mo, resulting in p-type doping. In the case of Nb substitution, the newly created impurity states arise mainly from the hybridized d orbitals of Nb and Mo (Figure 3a) and the excess charge propagates up to the third nearest neighbor Mo atoms [61]. The donor states have a similar character for the Zr and Y heteroatoms, but they are less hybridized [59]. It was shown that, in contrast to Re, the Nb dopant and the S vacancy do not tend to be neighbors [60]. Moreover, the doping properties of Nb are not sensitive to the presence of sulfur vacancies. Since these vacancies are formed during synthesis, the introduction of Nb into the MoS_2_ layers is promising for p-type doping of the material.

Consideration of a MoS_2_ supercell with more than one Nb showed a tendency for heteroatoms to stay close to each other. The preferred formation of a triatomic cluster centered on a hole of a hexagon (Figure 3b) was established for the heteroatoms Nb, Ta, Tc, and Re [62]. This behavior was related to easily accommodate the optimal atomic spacing for inter-dopant bonding. The formation energy calculated by Equation (2) was fairly insensitive to the concentration of heteroatoms in the range from 4 to 33%. It was shown that grouped heteroatoms change the local chemistry of the material and, therefore, are not efficient for n- or p-type doping as compared to isolated heteroatoms.

A screening of several earth-abundant metals Ti, V, Cr, Mn, Fe, Co, Ni, Zn, Zr, Nb, Hf, Ta, W, and Bi showed most metals prefer to be located at the S-edge, and only Nb, Zr, Hf, and Ta were better stabilized at the Mo-edge [40]. The tendency to turn on at the edges creates a problem for the synthesis of basal-plane doped MoS_2_. The examination of five configurations of the Fe atom in the MoS_2_ monolayer showed that this could be achieved under S-rich conditions [63]. 

The introduction of a foreign metal into the MoS_2_ layer is necessary to create a larger number of active sites per host unit and improve the electrical conductivity [40]. DFT calculations of Ni heteroatoms in bulk 2H-MoS_2_ revealed an increased conductivity due to band gap narrowing [64]. Interestingly, Ni can also easily replace sulfur. This means that Ni can fill existing S vacancies, which could be a way to synthesize Ni-doped MoS_2_. The results on the formation energy of the Ni heteroatom in the MoS_2_ monolayer obtained by Equation (3) showed that the filling of the S vacancy requires energy, while the filling of the Mo vacancy releases energy. The Ni heteroatom in the position of the Mo atom is energetically attractive for the formation of a sulfur vacancy in close proximity [65]. Easy movement of a heteroatom adsorbed above the Mo atom in MoS_2_ to fill a sulfur vacancy was also shown for Co [66].

#### 2.1.2. Sulfur Replacement

Molybdenum dichalcogenides MoCh_2_ (Ch = S, Se, Te) have a similar crystal structure and are, therefore, attractive for mixing in one compound. DFT relaxation of the structure of MoS_2(1−x)_Se_2x_ and MoS_2(1−x)_Te_2x_ alloys showed a linear behavior of the lattice constant with x (the Vegard’s law) [67]. The negative formation energy obtained for MoS_2(1−x)_Se_2x_ over the entire range 0 ≤ x ≤ 1 indicates complete miscibility of MoS_2_ and MoSe_2_ at low temperatures. The most thermodynamically stable alloys are those with x = 0.33, 0.5, and 0.66. In these structures, clustering of S or Se atoms is unfavorable, and two different chalcogens alternate around the Mo atom to maximize the number of dissimilar atom pairs (S–Se) [68]. The charge transfer from Se to the nearest S atoms ensures the energy gain from mixing MoS_2_ and MoSe_2_ [45]. Such charge transfer always exists whether the alloy is ordered or disordered, but it can be maximized by local S–Mo–Se ordering. MoS_2(1−x)_Se_2x_ monolayers retain a direct bandgap, the value of which decreases almost linearly with increasing x [67]. Mixing MoS_2_ with MoSe_2_ is more attractive than MoS_2_ with WS_2_ since the band gap in MoS_2(1−x)_Se_2x_ can vary in the range of about 0.3 eV, which is larger than the value of about 0.15 eV for Mo_(1−x)_W_x_S_2_ [69].

The formation energy of MoS_2(1−x)_Te_2x_ monolayers is positive, which indicates the absence of a stable configuration for these alloys at 0 K and phase separation in the alloys [67]. However, low values for the formation energy of a random configuration show that such alloy structures can be obtained at experimentally achievable temperatures. The calculated phase diagram showed an asymmetry that increased with temperature. The asymmetry means that, at a giving temperature, the introduction of Te atoms into MoS_2_ occurs more easily than S atoms into MoTe_2_. Full miscibility of MoS_2_ and MoTe_2_ is expected at about 493 K. The bandgap of random MoS_2(1−x)_Te_2x_ showed bowling behavior with deviation from linearity decreasing at x = 0.5. The change in the bandgap was about 0.4 eV in the range 0 ≤ x ≤ 1, which is greater than that calculated for MoS_2(1−x)_Se_2x_. In addition, substitutional Te atoms led to a larger localization of electrons near the valence band edge than Se atoms [70]. Replacing the entire S layer with Se or Te layers is possible under Mo-rich conditions, and replacing S with Se is preferred over Te due to its larger ionic size (2.1 Å vs. 1.9 Å for Se).

The calculation of MoS_2(1−x)_Te_2x_ monolayers with different Te distributions showed that configurations with ordered Te lines are thermodynamically stable at 0 K and compete with random alloys [71]. Configurations where the Te lines are far apart (to avoid clustering) have the lowest energy at each probed concentration x = 0.1, 0.3, 0.5, 0.7, and 0.9. The band gap depends on the composition of the alloy and covers the range of the visible spectrum.

The formation of line-ordered MoS_2_-based alloys was also predicted when sulfur was replaced by oxygen [72]. Oxygen belongs to the same group of the periodic table as the chalcogens and has the smallest atomic radii. It was shown that at large x in MoS_2−x_O_x_, line-ordered O alloys are more stable than random and cluster alloys, while at small x all configurations compete. The location of an equal number of O atoms in both layers sandwiching a Mo layer is energetically favorable for random alloys. All considered MoS_2−x_O_x_ structures retain the semiconducting features of MoS_2_ and the band gap decreases with increasing x. At the same O content, line-ordered alloys have a smaller bad gap than alloys with a random and cluster distribution of O. 

Halogens F, Cl, Br, and I have one additional valence p-electron with respect to S and should act as an n-type doping source for MoS_2_ [59]. In the example of a single Cl substituent, it was shown that the impurity states that appear at the edge of the conduction band arise as a result of hybridization between the Cl 3p and Mo 4d orbitals. Group V nonmetals of the periodic table (N, P, and As) have one less valence electron than sulfur. Replacing S with these elements in the concentration of 2% leads in the formation of impurity states above the valence band. In the case of As, these states merge with the valence band; therefore, this element is most promising for p-type doping [54,59]. An analysis of the density of states of the N-MoS_2_ monolayer indicated that the impurity states mainly arise as a result of hybridization between the N 2p orbitals and the 4d orbitals of the neighboring Mo [73]. The band gap narrows with an increase in the amount of N dopants and the monolayer becomes metal at a doping level of 12.5% [74].

C, Si, and Ge from group IV of the periodic table have a deficit of two valence electrons as compared to S. The introduction of 2% these heteroatoms into the MoS_2_ lattice leads to the appearance of impurity states far from the valence band, which makes them unsuitable for effective p-type doping [54]. The number of electrons is further reduced for group III elements, such as B, Al, and Ga. Dopant B induces impurity states closest to the valence band than any other elements from groups III and IV. It was concluded that elements P, As, and B are best suited for introducing gap states near the edge of the MoS_2_ valence band. Among these elements, the formation energy of B-MoS_2_ calculated by Equation (2) is smallest. However, for all elements of groups III–V, the formation energy has a positive value, which indicates they are unstable dopants at thermal equilibrium. The value decreases for the Mo-rich system [59]. Such conditions can occur at the Mo-terminated edges [40,75] or when sulfur vacancies are created in the layer. 

S-vacancies can be created by irradiating MoS_2_ with 80 eV electrons, as shown theoretically and confirmed experimentally [76]. The possibility of filling a monoatomic S-vacancy with isovalent atoms (O, S, Se, and Te), donors (F, Cl, Br, and I), acceptors (N, P, As, and Sb), hydrogen and group IV elements (C and Si) was checked by calculating the formation energy of a heteroatom in a MoS_2_ monolayer according to Equation (3). It was found that all substitutions are energetically favored with respect to isolated atoms. Calculations for C, Si, and N with respect to the chemical potential of CH_4_, SiH_4_, and NH_3_ molecules gave positive values. However, substitution by these elements can be achieved under an electron beam that destroys the molecule. 

**Figure 4:**
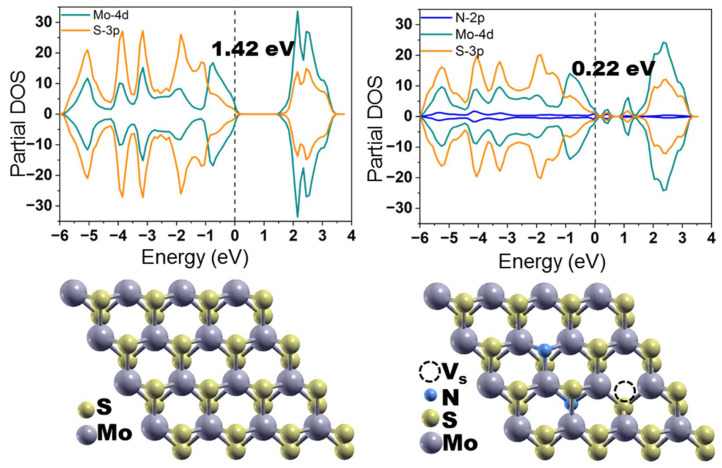
Partial density of states (**top figures**) calculated for ideal MoS_2_ monolayer and that with two substitutional N atoms and monovacancy (**models in bottom**). Dashed vertical lines correspond to the top of valence band, the numbers specify band gap values. Taken with permission from [78]; Copyright 2023, Elsevier B.V.

For phosphorus, it was obtained that an increase in the dopant concentration from 2% to 6% reduces the formation energy of the P-MoS_2_ monolayer [77]. The charge density is accumulated around the P dopant and the unoccupied P-3p_z_ orbital provides an active site for adsorbates, for example, hydrogen. A similar situation was observed for the N dopant due to charge transfer from less electronegative atoms Mo and S to its vicinity [73]. Among the calculated N-doped MoS_2_ monolayers, the lowest formation energy was obtained when N dopant was located near the S vacancy. The combination of a p-type dopant (substituent N) and impurity states induced by the S-vacancy near the conduction band led to a significant narrowing of the band gap of the MoS_2_ monolayer with such a defect complex (Figure 4) [78].

Easy filling of S-vacancies present in the MoS_2_ monolayer was also predicted for Cu and Ag [79] and Ge [80]. Cu and Ag have a much lower electronegativity (1.90 and 1.93) than sulfur (2.58), and, so their introduction in the layer causes a redistribution of charge on neighboring atoms [79]. As a result, polarized regions appear, activating the MoS_2_ surface for interaction with various chemical species. Unlike Cu and Ag, which have the lowest formation energy of a single heteroatom, Ge prefers to form a triatomic cluster [80]. The Ge dopants introduce new states in the band gap, thus enhancing the catalytic activity of MoS_2_ in oxygen reduction reaction. 

#### 2.1.3. Dual Replacement in MoS_2_

Simultaneous replacement of a part of molybdenum and sulfur atoms in MoS_2_ can be a way to fine tune the band gap, improve the thermodynamic stability of the compound due to an increase in entropy, and a stronger electron density redistribution along the layer. The isostructural nature of hexagonal MoS_2_, WS_2_, MoSe_2_, and WSe_2_, as well as the negative formation enthalpies of ternary alloys with isovalent cations and isovalent anions, make these disulfides and diselenides the most suitable for creating quaternary mixed compounds [45]. The DFT calculation of 152 random Mo_1−x_W_x_Se_2−y_S_y_ structures with x = y showed a continues band gap variation from 1.60 to 2.03 eV [81]. On the contrary, for the ternary compounds MoSe_2−x_S_x_ and Mo_1−x_W_x_S_2_, the band gap varies in narrow ranges 1.62–1.86 eV and 1.87–2.03 eV, respectively [49]. The largest change in band gap was found in Mo_1−x_W_x_Se_2(1−x)_S_2x_ at x = 0.5 [81]. Quarterly alloys demonstrated a great sensitivity of the electronic structure to the distribution of elements in the lattice.

Quarterly monolayer structures were created by replacing the Mo atom in MoS_2_ with Nb and the S atoms surrounding the substitutional Nb with one to six Se or Te atoms [70]. The Nb concentration was 2.1%, the concentration of chalcogen heteroatoms varied from 2.1 to 12.5%. It was shown that the band gap of MoS_2_ containing Nb substituents with six Se neighbors is larger than that of the ternary analogue without Nb. The opposite behavior (band gap reduction) was found in the case of Te. The substitutional Nb atom generates acceptor levels, resulting in p-type doping of quarterly monolayers. These levels are more localized in cases with substitutional Te than in those with substitutional Se. However, calculations revealed difficulties with the formation of these quarterly compounds because Nb incorporation is more favorable under S-rich conditions, while chalcogens require Mo-rich conditions. 

### 2.2. Experimental Detection 

Data on experimentally realized heteroatom doping of MoS_2_ were summarized in recent reviews [35,36]. Most of the elements considered in theoretical studies were successfully incorporated into the MoS_2_ structure experimentally (highlighted in red in the table in Figure 1). However, s-block metals tend to intercalate between MoS_2_ layers instead of replacing Mo, which is considered theoretically [39], while F and Cl atoms are more likely to be chemically adsorbed on the surface of MoS_2_ than to replace sulfur. 

**Figure 5:**
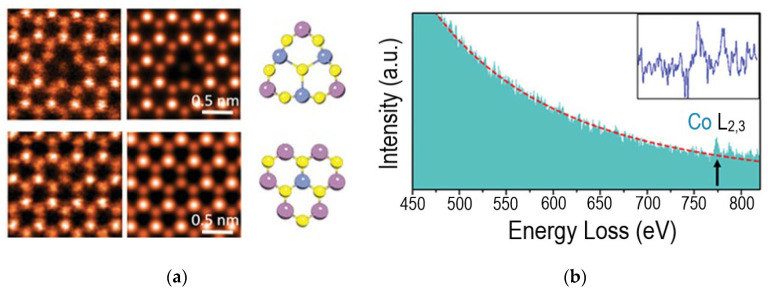
(**a**) Experimental (**left column**) and simulated (**right column**) atom-resolved STEM images for triatomic Co cluster (**top row**) and isolated Co atom (**bottom row**) replacing Mo atoms in a MoS_2_ monolayer. Purple, blue, and yellow balls in the models correspond to Mo, Co, and S, respectively. (**b**) EELS spectrum taken from places presented in (**a**), the arrow shows the Co L_2,3_ edge, enlarged in the inset. Taken with permission from [82]; Copyright 2020, John Wiley and Sons.

Direct methods for visualizing foreign atoms in the MoS_2_ lattice are high-resolution transmission electron microscopy (HRTEM) and high-angle annular-dark field/scanning transmission electron microscopy (HAADF/STEM). A clear demonstration of the possibilities of these methods for studying substitution-induced structural changes of MoS_2_ is given in [36]. However, it is not always possible to distinguish elements with similar contrast level. In this case, electron energy loss spectroscopy (EELS) at the single-atom level can help identify the element and its local atomic configurations [82,83]. Studies showed that some elements tend to form clusters, among them Co [82], Te [84], Re with a concentration below 1 at% [85,86], Se [87], W [88,89], Mn [90], Mg [91], Sb [92], and Ru [93] are distributed randomly regardless of concentration. Figure 5a shows an example of the identification of two different Co defects in a MoS_2_ monolayer by the HAADF/STEM method. Co and S have similar contrast levels and the incorporation of Co was confirmed by the Co L_2,3_ edge detection in the EELS spectrum (Figure 5b). The images simulated for models with a triatomic Co cluster and an isolated single Co substituent well agree with the experimental images (Figure 5a). 

The concentration and oxidation state of elements are usually determined using X-ray photoelectron spectroscopy (XPS). Some papers did not report binding energy calibration, making it difficult to compare their results with others. Calibration using the Au 4f_5/2_ peak and the Fermi level gives the position of the Mo 3d_5/2_ component at about 229.7 eV and the S 2p_3/2_ component at about 162.6 eV for 2H-MoS_2_ [94]. Baker et al., showed that the binding energy of the Mo 3d_5/2_ component decreases with sulfur depletion in the compound, and the difference between the Mo 3d_5/2_ and S 2p_3/2_ positions can be used to determine the stoichiometry of the MoS_x_ sample [95]. Doping causes a shift of the Fermi level of the compound, resulting in a change in the binding energy of the core-level electrons as compared to the undoped compound [96]. For example, a decrease in the binding energy of the Mo 3d_5/2_ and S 2p_3/2_ components was observed for MoS_2_ doped with Mg [91], Ru [93], N [97], and Nb [98,99], while the introduction of Re [86] or Se [87] substituents resulted in an increase in the binding energies. 

One of the most widely used methods for characterizing MoS_2_ nanomaterials is Raman spectroscopy. In-plane and out-of-plane S–Mo–S vibrations in the hexagonal MoS_2_ layer produce an E^1^_2g_ peak at ~382 cm^−1^ and an A_1g_ peak at ~407 cm^−1^, respectively [100]. The difference in the ionic radii of molybdenum, sulfur, and the substitutional elements can lead to the appearance of disorder, compressive stresses in the lattice, and changes in Mo–S distances. The E^1^_2g_ mode is more sensitive to these lattice modifications than the A_1g_ mode, which causes an increase in the separation of the corresponding Raman peaks of heteroatom-doped MoS_2_, as was observed for Se [101,102] and W [103,104]. Modifications of the MoS_2_ lattice can also be tracked by changes in the position and intensity of reflections on X-ray diffraction (XRD) patterns [99,105].

Distances between atoms in MoS_2_ containing heteroatoms can be determined using extended X-ray absorption fine structure (EXAFS) spectroscopy. EXAFS also defines a few coordination spheres of the specified element and types of neighbors [106]. The data of this method allow determining that foreign atoms are included in the host lattice and do not form separate clusters on its surface. For example, the study of Ni-doped MoS_2_ detected Ni–S bonds and no Ni–Ni bonds, indicating the atomic dispersion of nickel in the nanomaterial [107]. The same results were obtained for Nb-doped [108] and Co-doped [66] MoS_2_ in consistence with the HRTEM or HAADF/STEM data, which provide very local information about the sample structure. 

## 3. Gas Sensors

Layered inorganic nanomaterials are attractive for the detection of chemical species due to their large specific surface area, semiconducting properties with an appropriated band gap, and strong surface activity. An overview of the application of these nanomaterials, including MoS_2_, in various types of gas-sensing devices can be found in [24,25]. A report on the development of MoS_2_-based sensors for detection of gaseous nitrogen dioxide NO_2_ is presented in [32]. The dependence of the charge transfer process on MoS_2_ morphology, phase composition, the presence of atomic vacancies and heteroatom impurities, and the formation of heterostructures with graphene derivatives and metal oxides was considered and deeply analyzed. The NO_2_ sensing mechanism using MoS_2_-based heterostructures and composites was discussed in detail in a recent review [109]. 

In this section, we consider chemiresistive MoS_2_ sensors whose response is determined by the change in resistance due to the adsorption of the molecule. First, we briefly present the influence of morphology and edge states on the detection limits of various gases with undoped MoS_2_ and theoretical explanations on the observed effects. Then, we concentrate on the effect of heteroatoms on the interaction of MoS_2_ with molecules, studied experimentally and theoretically.

### 3.1. Undoped MoS_2_

The first sensor device was fabricated in 2012 from mono- and few-layered MoS_2_ films mechanically detached from a single crystal [110]. These exfoliated films were n-type doped and their resistance increased when exposed to NO gas, indicating charge transfer from MoS_2_ to the adsorbate. Films consisting of two to four MoS_2_ layers exhibited high sensitivity to NO with a detection limit of 0.8 ppm (parts per million) at room temperature, while the response of the MoS_2_ monolayer was fast but unstable. The devices showed a slow complete desorption of adsorbates, which was related to the strong interaction between MoS_2_ and NO. DFT calculations of NO adsorption on the surface of monolayer, bilayer, and trilayer MoS_2_ showed week physical adsorption in all cases with an energy gain of less than 215 meV [111]. NO adsorption induces impurity states near the bottom of the conduction band, which increases the conductivity of the MoS_2_ layers. This improvement is greater for a monolayer; however, few-layered MoS_2_ exhibited a higher electron mobility and this may be the determining factor for better performance of a sensor made of few layers of MoS_2_.

The following year, four papers on MoS_2_ sensors were published at once. Late et al., confirmed that few layers of mechanically delaminated MoS_2_ have a more stable response to NO_2_ and NH_3_ as compared to a monolayer [112]. The resistance of the devices decreased when exposed to NH_3_, which is an electron donor. Yao et al., fabricated a sensor device from few-layered nanosheets obtained by exfoliating bulk MoS_2_ crystal in an ethanol/water mixture [113]. The sensor was able to detect 5 ppm NH_3_ in ambient conditions. Significantly higher sensitivity to gaseous NH_3_, below 300 ppb (parts per billion), was achieved using the MoS_2_ films obtained by chemical vapor deposition (CVD) method via sulfurization of sputtered Mo thin layers [114]. Perkins et al., demonstrated the high sensitivity of a mechanically detached MoS_2_ monolayer to trimethylamine N(CH_2_CH_3_)_3_ and acetone(CH_3_)_2_CO vapors [115]. These molecules are electron donors and electron acceptors, respectively, and their adsorption on the MoS_2_ surface led to the opposite change in the device conductivity. The detection limit of the MoS_2_ monolayer device was ~1 ppm for N(CH_2_CH_3_)_3_ and ~500 ppm for (CH_3_)_2_CO at room temperature. 

The use of a CVD-grown MoS_2_ monolayer as the sensing element allowed reducing the detection limit of NO_2_ gas to 20 ppb and NH_3_ gas to 1 ppm at room temperature [116]. This study confirmed the higher sensitivity of the MoS_2_ basal plane to NO_2_ than to NH_3_ previously predicted based on adsorption energies obtained using DFT calculations [112]. The extremely large sensitivity of the sensor was associated with the large surface area of the monolayer, and it was proposed that the detection limit can be further reduced by optimizing the device configuration [116]. Approximation of the experimental data on the dependence of the sensor response to the concentration of the analyte determined the NO_2_ detection limit of 1.4 ppb for the MoS_2_ monolayer, which was synthesized by sulfurization of the MoO_3_ layer [117]. The sensor response was several times greater for 100 ppm NO_2_ as compared to the same concentration of H_2_S, methanol, SO_2_, and CO. 

The results of studying the interaction of NO_2_ and NH_3_ molecules with a defect-free MoS_2_ monolayer by DFT methods are summarized in [118]. All studies agree that the interaction occurs due to vdW forces, and there are several different configurations of molecules with nearly identical adsorption energy and charge-transfer characteristics. Such behavior may be critical for sensor functionality because the response is not highly dependent on the specific location or configuration of the adsorbate. 

MoS_2_ edges are more reactive that the basal plane [119,120]. This fact was used by Cho et al., to detect NO_2_ and ethanol [121]. Films with different orientation of the MoS_2_ layers were synthesized by sulfurization of pre-deposited Mo seed layers. The MoS_2_ layer growth direction changed from horizontal to vertical with respect to the substrate surface as the thickness of the Mo layer increased from 1 to 15 nm. The vertically aligned MoS_2_ layers exhibited responses enhanced by four and five times as compared to the horizontally oriented MoS_2_ layers for electron donor ethanol and electron acceptor NO_2_ molecules, respectively. The electrical response of the device correlated directly to the density of the exposed edged S. Detection limit determined for NO_2_ was 0.1 ppm. DFT calculations confirmed stronger binding of NO_2_ to the edge sites (Mo or S-terminated) as compared to the MoS_2_ basal plane sites. Edge-enriched MoS_2_ films were obtained by CVD method via sulfurization of MoO_3_ powder [122]. The films exhibited stable and reproducible responses to 2 ppm NH_3_ at room temperature and 20 ppb NO_2_ at a sensor operation temperature of 100 °C. Networks of MoS_2_ nanowires with abundant edges showed a low NO_2_ detection limit of 4.6 ppb at 60 °C [123]. Annealing the device in vacuum desorbed the oxygen-containing molecules from the MoS_2_ surface, thereby increasing the availability of the active edge sites for NO_2_ adsorption. 

Mechanically detached MoS_2_ monolayers were used to study the change in electrical conductivity upon adsorption of H_2_ at 200 °C [124]. It was shown that H_2_ molecules donate elections to MoS_2_, and the device is capable of detecting H_2_ concentration down to 0.1%. Based on the DFT calculations, it was suggested that the cause of electron donation is the dissociative adsorption of H_2_ on sulfur vacancies [125]. A sensor element made of vertically edge-exposed MoS_2_ flakes was able to detect 1% H_2_ at 28 °C [126]. DFT calculations revealed the highest probability of H_2_ adsorption at the Mo atom sites of the MoS_2_ edges. The Mo-terminated edges predominate in sulfur-deficient MoS_2_ [127].

High-performance humidity sensor was prepared from sonication-based exfoliated MoS_2_ flakes [128]. In wet air, the electrical conductivity of the sensor increased and changed by six orders of magnitude in the range of the relative humidity 10–95%. O_2_ gas was shown to act as an electron acceptor relative to the MoS_2_ sensor made from mechanically detached few-layer flakes [129]. The adsorption/desorption of O_2_ enhanced with applying a positive/negative voltage. DFT calculations showed a weak adsorption energy of 79 meV for O_2_ and 110 meV for H_2_O on the surface of a MoS_2_ monolayer [130]. The adsorbed molecules take 0.04e and 0.01e, respectively, from MoS_2_. Adsorption on sulfur-vacancy sites increases the charge transfer per molecule by a factor of five.

Nanospheres composed of thin MoS_2_ sheets grown in a hydrothermal process was used a sensor for the electron acceptor CO gas and showed a detection limit of 50 ppm at 175 °C [131]. The high response was attributed to point defects at the sheet edges. Indeed, according to DFT calculations, CO molecules strongly bind to unsaturated Mo atoms [132]. 

A comparative study of the adsorption properties of the MoS_2_ monolayer with respect to CO, CO_2_, NH_3_, NO, NO_2_, CH_4_, H_2_O, N_2_, O_2_, and SO_2_ with the DFT revealed a stronger binding for NO, NO_2_, and SO_2_ molecules [133]. Higher binding energy is usually associated with higher sensor sensitivity, since the MoS_2_ detection mechanism is based on charge transfer [134]. In addition, the adsorption of NO, NO_2_, and SO_2_ induces additional states near the edge of the conduction band of MoS_2_, while the other considered molecules have little effect on the band gap of MoS_2_ [133]. Thus, the basal plane of undoped MoS_2_ is most suitable for the detection of NO, NO_2_, and SO_2_ gases. The calculation of current-voltage curves revealed higher current for a bilayer MoS_2_ with NO_2_ as compared to a monolayer sensor [135].

### 3.2. Heteroatom-Doped MoS_2_

The significantly higher response observed experimentally for NO_2_ as compared to other gases [117,122,123,136,137] may indicate that the MoS_2_ sensor is selective to this toxic gas. Above, we demonstrated that sensor performance can be improved at elevated operation temperature [116,122,124,126] and by creating active sites such as edges [121] and atomic defects [126,131]. Heteroatom substituents are potential candidates not only for increasing the sensitivity of the sensor, but also for making it selective.

#### 3.2.1. Experimental Data 

**Figure 6:**
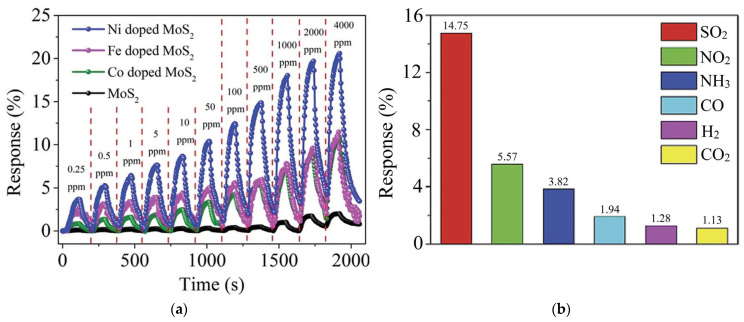
(**a**) Response of undoped MoS_2_ and doped MoS_2_ sensors toward varying concentrations of SO_2_; (**b**) selectivity of Ni-doped MoS_2_ sensor for 500 ppm of various gases; taken with permission from [141]; Copyright 2017, The Royal Society of Chemistry.

In a review on molybdenum-based gas sensors published in 2021, the replacement of sulfur or molybdenum with foreign atoms was considered as one of the strategies to improve the ability of MoS_2_ to detect gases [138]. However, the review mentioned only three experimental works on this topic published up to that time. Shao et al., synthesized ultrathin Zn-doped MoS_2_ nanosheets using a hydrothermal process with a zinc content ranging from 1 to 15% [139]. At room temperature, the chemiresistive sensor with 5% Zn showed a significantly higher response to O_3_ and NO_2_ as compared to undoped MoS_2_ synthesized by the same method. The detection limit of the 5% Zn-MoS_2_ sensor was 17 ppb for O_3_ and 215 ppb for NO_2_ at room temperature. Taufik et al., irradiated solvothermally prepared MoS_2_ nanoparticles with O_2_ plasma to replace sulfur by oxygen [140]. This treatment made the MoS_2_-based nanomaterial more sensitive to humid air in the range of 50–95%. The response to 95% humidity was much higher than the response to 100 ppm of interfering gases (acetone, ethanol, and oxygen), suggesting the applicability of the developed nanomaterial for a breath sensor. Zhang et al., used a hydrothermal route to introduce Ni, Fe, or Co into layers of MoS_2_ flower-like nanoparticles [141]. The doped sensors performed better for SO_2_ gas from 0.25 to 4000 ppm than undoped MoS_2_ (Figure 6a). The highest response was observed with the Ni-doped MoS_2_ sensor with a detection limit of 250 ppb at room temperature. The DFT calculations performed in this work confirmed that the SO_2_ molecule interacts more strongly with the nickel atom inserted into the MoS_2_ monolayer than with the inserted iron and cobalt atoms. The Ni-doped sensor exposed to 500 ppm of SO_2_, NO_2_, NH_3_, CO, CO_2_, and H_2_ at room temperature demonstrated a significantly higher response to SO_2_ (Figure 6b). Using a similar hydrothermal synthesis but with different precursors, Bharathi et al., synthesized flower-like nanoparticles composed of MoS_2_ layers with a nickel content ranging from 0 to 7 at% [142]. At room temperature, a sensor made from 7% Ni-MoS_2_ showed a higher response to NO_2_ gas than undoped MoS_2_. In contrast to a previous work, where the Ni-doped MoS_2_ sensor was selective to SO_2_ (Figure 6b), the 7% Ni-MoS_2_ showed NO_2_ selectivity among other tested gases H_2_S, NH_3_, CO, N_2_O, CO_2_, and SO_2_. Bharathi et al., suggested that in their nanomaterial, Ni atoms are located above the MoS_2_ layer, rather than replacing Mo atoms in the lattice. Unfortunately, in both compared works, the arrangement of Ni atoms was not proven by the HRTEM and/or EXAFS methods. 

**Figure 7:**
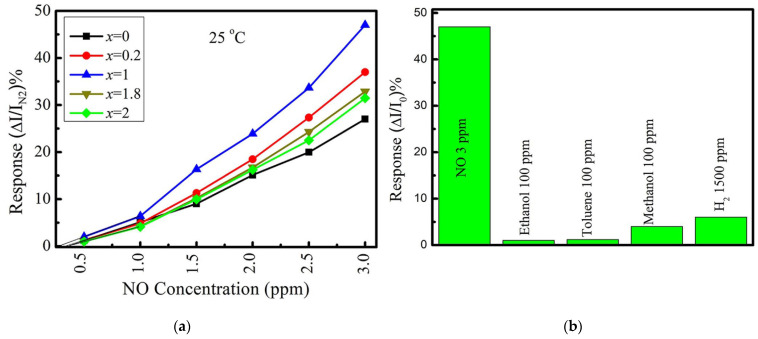
(**a**) Response of MoS_2−x_Se_x_ (x = 0, 0.2, 1, 1.8, 2) sensors for varying concentration of NO; (**b**) Selectivity of MoSSe sensor toward various gases; taken with permission from [143]; Copyright 2021, American Chemical Society.

Taufik et al., used hydrothermally synthesized MoS_2−x_Se_x_ (x = 0.2, 1.0, and 1.8) alloy nanoparticles to fabricate a room-temperature NO gas sensor [143]. The EXAFS study revealed the coexistence of Mo–S and Mo–Se bonds in the alloys. The MoSSe sensor showed a higher response as compared to other alloys and MoS_2_ and MoSe_2_ (Figure 7a) with a detection limit of 270 ppb. DFT calculations identified that NO tilted by the nitrogen atom for the surface of MoS_2_, MoSe_2_, and MoSSe monolayers, and in the latter case, the distance is shorter. In addition, the highest charge transfer was found for the NO + MoSSe system, which may be the reason for better detection performance of the MoSSe sensor. The response of this sensor to NO was stable under various humidity conditions and significantly higher than to ethanol, toluene and methanol vapors, and gaseous H_2_ (Figure 7b). Wu et al., synthesized N-doped MoS_2_ nanosheets with nitrogen contents of about 7, 10, and 13 at % using a modified solvothermal route [144]. The formation of Mo–N bonding was observed according to XPS data. Nanomaterials were used to detect NO_2_ gas at room temperature. All N-doped sensors demonstrated faster response and recovery as compared to undoped MoS_2_. The 10% N-MoS_2_ sensor, which showed the highest response among the examined devices, was taken to systematically evaluate overall sensing performance. This sensor was able to detect 125 ppb NO_2_ and at 10% relative humidity and it was highly selective for this gas among other CH_4_, NH_3_, H_2_S, and CO analytes. DFT calculations revealed that the NO_2_ molecule is located closer to the MoS_2_ monolayer with substitutional nitrogen than to the ideal monolayer, and the adsorption energy is larger in the former case. 

Ramaraj et al., compared the performance of Nb-doped MoS_2_ and MoS_2_ monolayers synthesized using a physical vapor deposition (PVD) technique, then transferred onto flexible polyethylene terephthalate substrates, and tested for NO_2_ gas detection at various operation temperatures [145]. Compared to undoped MoS_2_, the Nb-doped sensor showed improved response over the NO_2_ concentration range of 5 to 16 ppm and more stable recovery. This behavior was associated with the higher adsorption energy of the NO_2_ molecule on the embedded Nb, obtained using DFT calculations. The response to 5 ppm NO_2_ was significantly higher than to the same concentrations of toluene, acetone, methane, and CO, demonstrating sensor selectivity at 50–200 °C. Zhang et al., synthesized large-areas undoped MoS_2_ and Nb-doped MoS_2_ monolayer films by the metal organic CVD method [146]. The XPS data determined about 6 at% Nb and an upshift of the valence band maximum for the latter film due to p-type doping. Since the type of doping of the films was different, they showed an increase (undoped MoS_2_) and a decrease (Nb-doped MoS_2_) in conductivity when exposed to trimethylamine. The Nb-doped sensor exhibited a 50-times-higher signal-to-noise ratio as compared to undoped MoS_2_. The signal was well detected by the former sensor at a trimethylamine concentration of 15 ppb. A more efficient charge transfer between the analyte and the Nb-doped sensor was associated with the proximity of the impurity states induced by Nb heteroatoms to the highly occupied molecular orbital of the adsorbed molecule. 

Table 1 summarizes the data on heteroatom-doped MoS_2_ gas sensors together with best undoped MoS_2_ sensors. The parameter being compared is the detection limit of the analyzed gas at a given operation temperature. The most tested gas is NO_2_ since the conductivity of MoS_2_ changes strongly after adsorption of NO_2_ molecules. The lowest detection limit of 1.4 ppb NO_2_ was achieved using a CVD-grown MoS_2_ monolayer at room temperature. Note that many sensors fabricated from undoped MoS_2_ nanomaterials have sub-ppm levels of NO_2_ detection, which is ensured by the introduction of edge and defect states into the lattice and/or an increase in the operation temperature of the device. The developed heteroatom-doped MoS_2_ sensors also detect sub-ppm concentrations of NO_2_, and N-doped MoS_2_ demonstrated the lowest detection limit. Heteroatom doping made it possible to significantly improve the response of the MoS_2_-based sensor to NO and N(CH_2_CH_3_)_3_ gases.

#### 3.2.2. DFT Calculation of the Adsorption Energy 

The role of DFT calculations in unlocking the potential of two-dimensional nanomaterials for gas sensing applications was discussed in review [147]. Calculations are needed to explain the experimental results, to deeply understand the mechanism of the gas detection, and to provide guidance on the design of nanomaterials that are sensitive and selective for a particular analyte. The interaction of a molecule with the sensor surface is explored by analyzing the adsorption energy, charge transfer, density of states, band gap structure, and geometric parameters for optimal adsorption configurations. The density of states and the band gap structure characterize the change in the electronic properties of sensor after gas adsorption. The main calculation parameter is the adsorption energy, which is, in the case of a MoS_2_ sensor, defined as:

E_ads_ = E_MoS2+mol_ − (E_MoS2_ + E_mol_), (4)

where the terms are the total energies of the optimized MoS_2_ structure with an adsorbed molecule, an adsorbate-free structure and a free molecule. A negative E_ads_ indicates that the adsorption process is exothermic and can occur at room temperature. The greater the energy value, the stronger the interaction between the components. Charge analysis is performed using the Mulliken, Hirshfield, Löwdin, or Bader schemes, and the analyzed value is the charge transfer (ΔQ) between the molecule and the substrate. A positive (negative) charge that appears on an adsorbed molecule means it donates (accepts) electron density to (from) the sensor. The sensor–adsorbate distance is an indicator of the nature of adsorption. If the distance is larger (less) than the sum of the radii of closely contacting atoms from the surface and the molecule, adsorption is physical (chemical). 

To characterize the interaction of heteroatom-doped MoS_2_ and a gas molecule, the three calculated parameters E_ads_, ΔQ, and the shortest sensor–adsorbate distance (d) are compared in Table 2. Only the results obtained using the generalized gradient approximation (GGA) for the exchange-correlation Perdew–Burke–Ernzerhof (PBE) functional with allowance for long-range interactions were considered. These interactions are especially significant in the case of physical adsorption and they were taken into account by the Grimme dispersion corrections D, D2, D3, or the vdW correction to the density functional [148]. In all cases, a MoS_2_ monolayer was considered, and a foreign atom was introduced into a sulfur vacancy.

**Figure 8:**
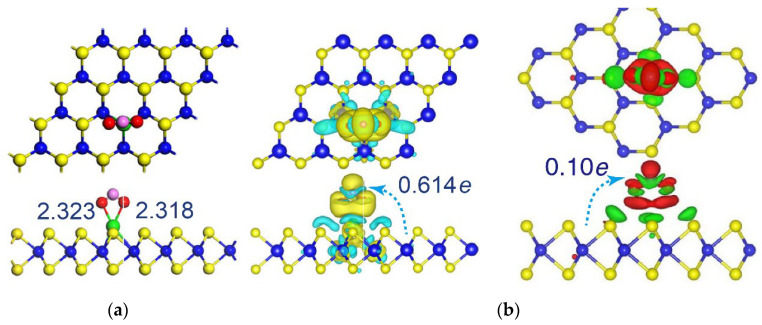
(**a**) Top and side view of NO_2_ located above Ag (green ball) substituent in MoS_2_. The values show the distances (in Å) between Ag and O atoms of NO_2_; taken with permission from [153]; Copyright 2023, AIP Publishing; (**b**) charge density difference plots and value of charge transferred for Ag-doped MoS_2_ (**left**) and undoped MoS_2_ (**right**) with adsorbed NO_2_. Blue (yellow) in left and green (red) in right correspond to depletion (excess) of electron density; taken with permission from [159]; Copyright 2013 by the authors.

DFT studies showed that the perfect MoS_2_ is not suitable for detecting gas molecules, while doping with a heteroatom usually has a positive effect. A systematic study of the effect of various transition metals (Fe, Co, Ni, Cu, Ag, Au, Rh, Pd, Pt, and Ir) embedded in a MoS_2_ monolayer on the adsorption of CO, NO, O_2_, NO_2_, and NH_3_ was presented in [149]. Foreign metals significantly modulate the electronic structure of MoS_2_, which enhances the adsorption of gas molecules. An important role in this is played by impurity states arising in the MoS_2_ band gap. Among the considered metals, Fe and Co are the best candidates for improving the sensor performance of MoS_2_. Szary investigated the sensitivity of MoS_2_ doped with transition metals Ti, Ni, Pd, and p-block elements Si, Ge, P, and Cl toward ethylene oxide (C_2_H_4_O) [150]. Ti, Ni, Pd, and Si atoms have been shown to facilitate the interaction of MoS_2_ with C_2_H_4_O, and a higher sensor response is expected for Si-doped MoS_2_. Deng et al., showed that undoped MoS_2_ is insensitive to the formaldehyde (CH_2_O) molecule, while its doping with Ni, Pt, Ti, and Pd leads to an increase in the adsorption energy and the largest gain is provided in the case of Ti [151]. Song and Lou considered the effect of Ag-doping on the interaction of a MoS_2_ monolayer with NO_2_, NH_3_, H_2_S, SO_2_, CO, and CH_2_O gases [153]. All molecules are chemically adsorbed on the Ag-doped surface by bonding with the foreign metal. Stronger adsorption was found for NO_2_. The molecule is oriented by oxygen atoms to the Ag substituent (Figure 8a) and in this case, the charge transfer to the adsorbate is six times higher than in the case of undoped MoS_2_ [159] (Figure 8b). In both cases, most of the transferred electron density was accumulated between substrate and NO_2_. According to [154], MoS_2_ doped with Ag is inert to NO gas, while the Au-doped MoS_2_ is sensitive. Co-doping of Au and Ag promises to create a highly sensitive MoS_2_-based sensor for NO_2_. MoS_2_ doped with V, Nb, or Ta was proposed to be suitable for detection of CO, H_2_O, and NH_3_ molecules [155], while Si-doped MoS_2_ could be a good sensor for SF_6_ [156]. 

The theory shows that doping of MoS_2_ with metals is, in general, a more efficient way to fabricate sensitive and selective sensors than doping with non-metals. Heteroatom-doped MoS_2_ donates electron density to CO, NO, NO_2_, and SO_2_ molecules and accepts it from NH_3_, C_2_H_4_O, SOF_2_, and H_2_O. The behavior, with respect to H_2_S and O_2_, depends on the type of heteroatom (Table 2). The highest binding energy is usually obtained for the NO_2_ molecule, which agrees with the experimental data. 

## 4. Electrochemical Energy Storage

The ever-increasing demand for renewable energy is driving the search for new, highly efficient energy storage materials. Today, LIBs are widely used in various portable devices, but the vector is shifting towards the development of SIBs and PIBs, which are particularly required in stationary energy storage installations and electric vehicles [160]. Supercapacitors complement rechargeable batteries, as they provide high powder density and have long life. MoS_2_ is attractive for energy storage applications due to the weak vdW coupling of the layers and the large distance between them. This facilitates the metal ion intercalation and diffusion and is especially important for large ions like Na^+^ and K^+^ [161]. There are many simple methods for the synthesis of MoS_2_, which allow varying the architecture and the number of adjacent layers in a nanomaterial [162], thereby adjusting its electrical conductivity and the density of active sites for ion adsorption, which is important for high electrode capacity and its stability. The main disadvantages of MoS_2_ is its low conductivity, which prevents rapid electron transfer that occurs in batteries and supercapacitors, and the tendency of layers to aggregate, which reduces ion diffusion and structure stability [163,164]. In addition, the electrochemical activity of MoS_2_ decreases due to the sluggish reaction kinetics [165].

Rational structural engineering of MoS_2_ nanomaterials by controlling the shape and phase, chemical doping, interlayer spacing, type and defect density, etc., leads to an increase in capacity, power capability, and cycle life of electrodes [165,166]. The role of defects in MoS_2_ performance in LIBs, SIBs, and PIBs is analyzed in [167]. The review emphasized that structural defects modify the chemical and electronic properties of nanomaterials, create more storage spaces for ions (thus enhancing capacity), reduce the stress between adjacent layers (which affects the insertion/extraction of ions), and promote charge transfer in electrochemical processes. 

We focused on the effect of substitutional doping MoS_2_ with heteroatoms on the performance of nanomaterials in LIBs, SIBs, PIBs, and supercapacitors. 

### 4.1. Lithium-Ion Batteries

The electrochemical interaction of MoS_2_ with lithium ions includes intercalation and conversion reactions. The intercalation reaction proceeds at potentials above 1.1 V vs. Li/Li^+^ and provides a capacity of 167 mAh∙g^−1^ in the formation of the LiMoS_2_ compound [10]. With further insertion of lithium ions into the interlayer space, the MoS_2_ lattice is destroyed with the formation of Mo clusters and lithium sulfide Li_2_S [168]. The reversibility of this reaction, i.e., the recovery of MoS_2_ after the extraction of lithium from the electrode material, is still the subject of discussion. The most common point of view is that the reaction is irreversible [169,170]; however, the studies of the reaction products sometimes reveal the retention of MoS_2_ layers even after several repeated intercalation/de-intercalation cycles [127,171,172]. Synchrotron X-ray absorption spectroscopy and Raman spectroscopy studies on lithiated and delithiated MoS_2_ electrodes concluded that the reaction may be partially reversible [173], and nanostructuring of MoS_2_ [127] and/or its hybridization with graphene [174] can contribute to its reversibility. 

The theoretical capacity of MoS_2_ provided by the intercalation reaction and the conversion reaction is 669 mAh∙g^−1^, due to the transfer of four electrons [10]. This value is almost two times higher than that for graphite, used as an anode material for commercial LIBs. After converting Li_x_MoS_2_ to Mo and Li_x_S_2_, the following electrochemical processes involve reversible reactions between sulfur and lithium ions similar to those that occur in lithium–sulfur batteries [175]. The theoretical capacity of sulfur for lithium ions is 1672 mAh∙g^−1^, and such values were observed for MoS_2_-based electrodes [176,177,178]. Note that the electrode material contains the heavy element molybdenum, and if we recalculate the specific capacity of the material only by the mass of sulfur, the experimental values of the capacity exceed the theoretical value. The enhancement may be related to the reversible interaction of Mo clusters with sulfur [179]. 

The main problem of MoS_2_ electrodes is the rapid capacity fading during LIB operation [161,180]. The reasons for this are the poor electrical conductivity of MoS_2_, the expansion of the lattice in the volume during intercalation, and the dissolution of lithium polysulfides Li_2_S_n_ (2 ≤ n ≤ 8) in organic electrolytes, which leads to a loss of electrochemically active sulfur. These disadvantages can be eliminated by hybridizing MoS_2_ with a carbon component, creating composites and heterostructures with other compounds, and inserting foreign elements into the MoS_2_ lattice. 

The first demonstration of the performance of the MoS_2_ material as a LIB anode is dated 2009 [181]; therefore, there are not so many works on the heteroatom-doped MoS_2_ anodes. Most of the research in this area is devoted to N-doped MoS_2_. Liu et al., synthesized MoS_2_ nanoparticles by a hydrothermal route and then heated them in a flow of Ar or NH_3_ at 700 °C for 2 h [182]. According to XPS data, the nitrogen content in the latter sample was ~5 at%. N-doped MoS_2_ showed an initial specific capacity of 1130.8 mAh∙g^−1^ at a current density of 0.2 C (134 mA∙g^−1^), which was ~1.2 times higher than that of undoped MoS_2_, and was able to reversibly deliver ~800 mAh∙g^−1^. This improvement was preserved at continues cycling, while both nanomaterials constantly lost their capacity. Based on electrochemical impedance spectroscopy (EIS) data, the authors concluded that doping of MoS_2_ with nitrogen significantly reduces the charge resistance in the electrode. DFT calculations for MoS_2_ and N-doped MoS_2_ monolayers revealed a narrowing of the band gap by about 0.2 eV in the latter case, which could provide faster electron and ion transport. Li et al., used a similar procedure to introduce nitrogen into the MoS_2_ layers, but the NH_3_ treatment temperature was 300 °C [183]. N-doped MoS_2_ electrode had an initial specific capacity of 1186 mAh∙g^−1^ at 0.1 C, which slowly but gradually decreased over next 100 operation cycles of the LIB. Jiao et al., synthesized flower-like nanoparticles from graphene oxide (GO) and MoS_2_ by a hydrothermal method and they then treated the composite with N plasma at room temperature to reduce GO (rGO) and introduce nitrogen into both components of the nanomaterial [184]. Plasma treatment greatly increased the capacity and improved the stability of the electrode as compared to undoped one. The initial specific capacity of N-rGO/MoS_2_ was 726.0 mAh∙g^−1^ at 1 C and the capacity retention after 100 cycles was 81.5%. Later, researchers from this group modified the synthesis procedure by adding L-cysteine as a spacer between the MoS_2_ layers [185]. This structural modification increased the initial capacity of N-rGO/MoS_2_ to 1725.6 mAh∙g^−1^ at 0.1 A∙g^−1^, which is 1.6 times larger than that of the composite nanomaterial without N plasma treatment. The N-rGO/MoS_2_ electrode showed stable operation in LIB for 400 cycles at a current density of 0.5 A∙g^–1^. Based on experimental and theoretical studies of N-doped graphene/MoS_2_ composites, Cho et al., found that both of these components provide high chemisorption energy for lithium polysulfides, which can increase life of the electrode [186]. 

Fayed et al., showed that the use of carbon-containing compounds in the hydrothermal synthesis of MoS_2_ nanomaterials actually leads to co-doping with nitrogen and carbon [187]. The initial specific capacity of the electrode was 1280 mAh∙g^−1^ at 0.1 A∙g^−1^; after 60 cycles, it decreased to about 400 mAh∙g^−1^.

Two works were devoted to the study of the effect of heteroatoms on the intercalation reaction of MoS_2_ with lithium ions. Kotsun et al., synthesized N-doped MoS_2_ plates by rapid thermolysis of ammonium tetrathiomolybdate (NH_4_)_2_MoS_4_ in an ammonia atmosphere and tested them in LIBs at potentials above 1.1 V [78]. The nanomaterial with a nitrogen content of about 4% had a specific capacity of 189 mAh∙g^−1^ at 0.05 A∙g^−1^, which is higher than the theoretical value for ideal MoS_2_. DFT calculations showed that in the interlayer space of the N-MoS_2_ bilayer, lithium prefers to bind with two nitrogen atoms from opposite layers. Even when lithium is far from the incorporated N, the adsorption energy is higher than that for an ideal bilayer. This behavior provides a higher capacity for N-doped MoS_2_. Gong et al., used a hydrothermal method to decorate a porous graphene aerogel with oxygen-incorporated MoS_2_ and investigated a self-supported electrode for lithium intercalation/de-intercalation in the potential range from 3 to 1 V vs Li/Li^+^ [188]. The electrode showed high rate performance and outstanding durability, retaining about 91% capacity at a current density of 2 A∙g^−1^ after 3000 cycles. 

**Figure 9:**
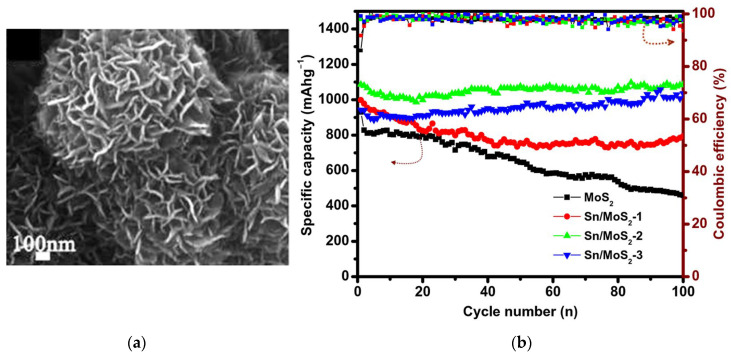
(**a**) Scanning electron microscopy image of 7.9% Sn-doped MoS_2_ flowers; (**b**) cycling performance of undoped MoS_2_ and MoS_2_ doped with 4.3% (Sn/MoS_2_-1), 7.9% (Sn/MoS_2_-2), and 14.9% (Sn/MoS_2_-3) of tin in LIBs at a current density of 0.2 Ah∙g^−1^; taken with permission from [189]; Copyright 2016, Springer–Verlag.

Lu et al., synthesized Sn-doped MoS_2_ flowers (Figure 9a) using SnO_2_ as the starting compound in a solvothermal synthesis [189]. Nanomaterials with a tin content of 4.3, 7.9, and 14.9 at% showed higher stability over 100 cycles as compared to undoped material (Figure 9b). The 7.9% Sn-doped MoS_2_ exhibited an excellent initial capacity of 1087 mAh∙g^−1^ at a current density of 0.2 A∙g^−1^, which remained constant after 100 operation cycles of the LIB. 

Lei et al., introduced 17.6% vanadium in MoS_2_ using vanadocene in a solid-state reaction [190]. The initial electrode capacity of 814 mAh∙g^−1^ at 1 A∙g^−1^ decreased to 350 mAh∙g^−1^ by the 300th cycle. The study of few-layer MoS_2−x_Se_x_ nanosheets synthesized using MoO_3_ nanowires as a skeleton structure, and a source of sulfur/selenium under hydrothermal conditions showed that x = 0.25 is optimal to provide a high reversible specific capacity of ~1077 mAh∙g^−1^ at 0.1 C in LIBs [191]. The incorporation of selenium not only improved the electrode capacity, but also accelerated the movement of lithium ions, which led to stable operation of the battery at 10 C.

**Figure 10:**
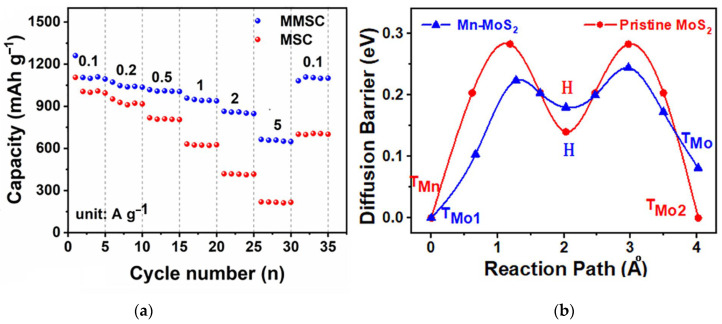
(**a**) Rate performance of undoped MoS_2_/carbon cloth (MSC) and Mn-doped (MMSC) electrodes in LIBs; (**b**) diffusion barrier calculated for Li atoms over Mn-doped MoS_2_ monolayer and pristine MoS_2_ monolayer; taken with permission from [194]; Copyright 2019, Elsevier B.V.

The most impressive performances were obtained when heteroatom-doped MoS_2_ was combined with a carbon component. Wang et al., grew mesoporous Se-doped MoS_2_ layers vertically to the surface of rGO and showed high rate capability and good cycling stability for the composite with MoS_1.12_Se_0.88_ [192]. From a comparison of the performance of this composite with and without the carbon component, the authors concluded that graphene improves the conductivity and flexibility of the electrode material and reduces its volumetric expansion during the discharge/charge of LIBs. Francis et al., grew P-doped MoS_2_ nanoparticles on carbon cloth using sodium hypophosphite as the phosphorus source in hydrothermal synthesis [193]. The nanomaterial was used as a binder-free anode in LIB and exhibited an initial capacity of 2700 mAh∙g^−1^ at a current density 0.1 mAh∙g^−1^, which decreased to 900 mAh∙g^−1^ after 50 operation cycles. The authors supposed that P–O bonds reduce the agglomeration of MoS_2_ nanoparticles. Wang et al., used the same synthesis method to attach Mn-doped MoS_2_ nanosheets on a hierarchical carbon cloth [194]. The Mn-doped electrode showed a higher specific capacity as compared to the undoped one, especially at high current densities (Figure 10a), which, according to DFT calculations, is explained by increased conductivity and a lower diffusion barrier for lithium along the Mn-MoS_2_ layer (Figure 10b).

Qi et al., obtained ultra-thin Fe-doped MoS_2_ nanosheets on rGO using iron sulfite in hydrothermal synthesis [195]. The role of iron in providing a high reversible specific capacity of 946 mAh∙g^−1^ at 0.1 A∙g^−1^ after 1000 operation cycles of LIB was associated to an increase in the density of lattice defects and a decrease in electrode resistance. The Co-doped MoS_2_/rGO nanomaterial, in which a Co atom replaced every fifth Mo atom, was able to deliver a specific capacity of 894 mAh∙g^−1^ at a current density of 1 mAh∙g^−1^ [196]. An increase in the cobalt content in MoS_2_ provided excellent rate performance of the Co_1/3_Mo_2/3_S_2_/graphene nanocomposite, which showed stable operation at a current density of 50 A∙g^−1^ [197]. Han et al., synthesized nanoparticles from N-doped graphene and layers of MoS_2_ doped with Fe, Co, or Fe, Co by thermolysis of a mixture of compounds containing these elements [198]. The inclusion of both metals (Fe and Co) in the composition of the nanomaterial provided the highest specific capacities in the range from 0.1 to 20 C and high electrode stability at a current density of 5 C for 3000 operation cycles. The FeCo-MoS_2_/carbon electrode was assembled with commercial LiFePO_4_ into a full LIB cell, which, after 200 cycles, delivered 127 mAh∙g^−1^ at 1 C, demonstrating a capacity retention of 91.8%. The magnetic atoms in the composite electrode ensured efficient separation of the MoS_2_ and carbon layers, high electrical conductivity, and fast reaction with lithium ions. 

Data on the initial specific capacity provided by heteroatom-doped MoS_2_ anodes in LIBs and the reversible specific capacity after several operation cycles are collected in Table 3. Among the dopants probed, nitrogen substitution gave the lowest values of specific capacity. Sn, Mn, and Co are very promising substituents, and the excellent performance obtained for FeCo-doped MoS_2_/carbon encourages the search for optimal combinations of dopants to ensure high rate capability, capacity retention, and life of the electrode material.

DFT calculations of lithium interaction with MoS_2_ monolayers containing Fe, Co, Ni, Cu, and Zn instead of Mo or N, P, As, F, Cl, and I instead of S revealed higher adsorption energies near the substituents as compared to undoped MoS_2_ [199]. Doping with heteroatoms does not significantly affect the barriers for lithium diffusion through the layer, except for positions near the substituent. 

### 4.2. Sodium-Ion and Potassium-Ion Batteries

Sodium and potassium belong to the same group of the periodic table of chemical elements as lithium and, therefore, have similar (electro)chemical properties. Thus, the methods of synthesis and characterization, as well as the design of electrochemical cells developed for LIBs, can be transferred to sodium and potassium analogues. Moreover, these chemical elements, due to their low cost, are alternative candidates for rechargeable batteries [160]. MoS_2_ possessing a large interlayer distance is a promising host for large ions and is, therefore, actively studied as anode materials for SIBs and PIBs [161,200]. The current understanding of the basic principles of charge storage in SIBs and PIBs can be found in [161]. In contrast to the lithiation process, in which the destruction of the MoS_2_ lattice occurs after the formation of the LiMoS_2_ compound, sodium intercalation is possible up to 1.75 Na per MoS_2_ structural unit [201]. Electrochemical intercalation of potassium ions can proceed until 0 V vs K/K^+^ [202], which is different from the cases in LIBs (~1.1 V vs. Li/Li^+^) and SIBs (about 0.8 V vs. Na/Na^+^).

Heteroatom-doped MoS_2_ nanomaterials are usually combined with a carbon component (Table 4) to improve the electrical conductivity and structural stability of the electrode material in the SIB. This component is introduced intentionally by adding graphene oxide [197,203] or a carbon source [198,204,205,206,207,208,209,210] in the synthesis or is formed by the decomposition of a carbon-containing solvent [206]. Wang et al., used a nitrogen plasma treatment of rGO/MoS_2_ for the doping [203]. Nitrogen is most often used as a substitutional element for MoS_2_ doping (Table 4), as it easily incorporates into the MoS_2_ lattice under hydrothermal conditions [204,205,208] or thermal decomposition of thioacetamide [206,207]. As a rule, the formation of the Mo–N bond is confirmed by the component located around 396.4–396.9 eV in the XPS spectrum. Sometimes, the choice of this component is not obvious, since the N 1s and Mo 2p_3/2_ regions overlap. In all cases, the authors observed a higher specific capacity of N-doped electrode as compared to undoped electrode prepared under similar conditions. This improvement is associated with an increase in the interlayer distance and conductivity of MoS_2_, which facilitates the transport of sodium ions [203,206,207]. For example, the diffusion coefficient of the ions in N-rGO/MoS_2_ was 1.64 times higher than that in the undoped counterpart [203]. Regardless of the synthesis protocol, the N-doped MoS_2_/carbon composites exhibited similar values of the reversible capacity after several hundred cycles of SIB operation (Table 4). Note that the carbon component increases the capacity, and its contribution depends on the ability to accumulate sodium ions, fractions in the composite, and interaction with MoS_2_. 

The substitution of sulfur for antimony induced the transformation of semiconducting 2H phase of MoS_2_ to metallic 1T phase [209]. The obtained Sb-doped MoS_2_/N-carbon electrode was able to deliver 183 mAh∙g^−1^ at 5 A∙g^−1^ and showed a superior long-term operation at 5 A∙g^−1^ (Table 4). The material was used to assemble a full SIB with Na_3_V_2_(PO_4_)_3_ cathode maintained a capacity of 242 mAh∙g^−1^ at 0.5 A∙g^−1^ after 100 cycles. After the same number of cycles, the full cell with the N-doped MoS_2_ foam anode showed only 143.6 mAh∙g^−1^ at 0.33 A∙g^−1^ [205]. Zong et al., prepared Te-doped MoS_2_ nanosheets on the surface of tubular carbon and then coated the composite with carbon [211]. The nanomaterial showed a high rate performance in SIB, delivering 255.1 mAh∙g^−1^ at a current density of 10 A∙g^−1^. The replacement of sulfur by tellurium was shown to increase the interlayer distance in MoS_2_ to 0.95 nm, cause the formation of the 1T phase, and promote the adsorption of Na^+^ ions. 

Woo et al., synthesized the carbon-free undoped and Re-doped fullerene-like MoS_2_ nanoparticles by treating MoO_3_ and Re_x_Mo_1−x_O_3_ (x < 0.01) with H_2_S in a reducing atmosphere at 800–870 °C [212]. Tests in the range between 0.7 and 2.7 V vs. Na/Na^+^, when intercalation occurs, showed better cycling performance of the doped electrode. Crystal defects and dislocations served as channels for the insertion of Na^+^ ions and the number of these channels was greater in Re-doped MoS_2_. After full desodiation of the electrode, the crystal structure of MoS_2_ was not restored, according to XRD data.

**Figure 11:**
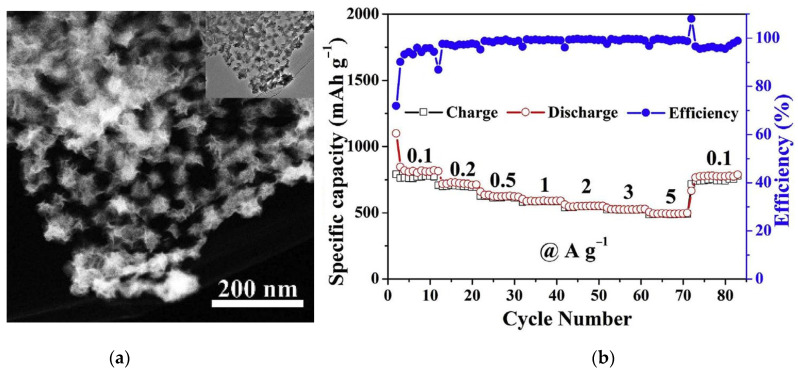
(**a**) HAADF/STEM image and HRTEM image (inset) of Co_1/3_Mo_2/3_S_2_/rGO; (**b**) cycling performance of Co_1/3_Mo_2/3_S_2_/rGO electrode at various current densities in SIB; taken with permission from [197]; Copyright 2018, Elsevier S.A.

Similar to N substituent, the effect from the incorporation of foreign metals in the MoS_2_ lattice is also considered as an enlarged interlayer spacing and improved conductivity of the electrode material. In the case of Mn-doped MoS_2_ nanotubes, this caused an increase in the Na^+^ diffusion coefficient by 2.9 times as compared to undoped nanotubes [213]. DFT calculations revealed that the substitution of every fourth molybdenum by a vanadium atom almost halves the energy barrier for the migration of sodium ions in the interlayer space [214]. This result was used by the authors to explain the excellent rate performance of VMoS_2_ electrode, which was stable at a current density of 20 A∙g^−1^. Furthermore, these authors modified the synthesis procedure to obtain the orderly layered VMoS_2_ [215]. The edges of stacked nanosheets provided additional storage sites for Na^+^ ions, and the anode showed a specific capacity of 497.5 mAh∙g^−1^ at 5 A∙g^−1^ with a Coulombic efficiency of up to 100% at the 490th cycle. A similar capacity value of 491 mAh∙g^−1^ at 5 A∙g^−1^ was obtained for the Co_1/3_Mo_2/3_S_2_/r-GO composite (Figure 11) [197]. The Ni-MoS_2_@porous carbon anode provided a reversible capacity of 420.8 mAh∙g^−1^ at 5 A∙g^−1^ and 386.6 mAh∙g^−1^ at 5 A∙g^−1^ [210]. The DFT calculations revealed that Ni substituent can significantly improve the adsorption of Na^+^. The alternation of Fe and Co-doped MoS_2_ layers and N and O-doped graphene layers in the nanomaterial provided high capacities ranging from 735.7 to 340.8 mAh∙g^−1^ at current densities from 0.1 to 20 C [198]. DFT calculations gave energy barriers of 0.26 and 0.14 eV for sodium migration in the space between graphene and MoS_2_ layers and graphene and metal-doped MoS_2_, respectively. Therefore, the charge redistribution in MoS_2_ caused by the Fe and Co co-doping accelerates the diffusion of ions. Moreover, the incorporation of foreign metals makes the bilayer metallic, which should facilitate the transport of electrons in the electrode. 

Potassium ion has a larger radii (0.138 nm) than sodium (0.102 nm); however, its low reduction potential (−2.93 V vs. the standard hydrogen electrode) indicates possible high energy density for PIBs [216]. Insertion of heteroatoms in the MoS_2_ lattice increases the interlayer distance [209,217,218], narrows the bandgap [216,219,220], and increases the diffusion coefficients of potassium ions [217,218,219,220,221]. The values of the initial specific capacity and reversible capacity during long-term cycling of heteroatom-doped MoS_2_ anodes are presented in Table 5. The performance depends on the nature and content of foreign element, the presence of carbon component, morphology, and phase composition. 

Liu et al., sulfurized a mixture of chitosan and phosphormolybdic acid at 600 °C to obtain ultrathin MoS_2_ nanosheets evenly embedded in a nitrogen-doped carbon matrix [209]. The addition of SbCl_5_ in the reaction mixture led to the formation of the 1T phase of MoS_2_ doped with Sb. The doped electrode showed outstanding potassium storage performance: 150 mAh∙g^−1^ at 1 A∙g^−1^ for 1000 cycles. The chitosan-derived carbon reduced the variation in the MoS_2_ volume upon the insertion/extraction of K^+^ ions. 

**Figure 12:**
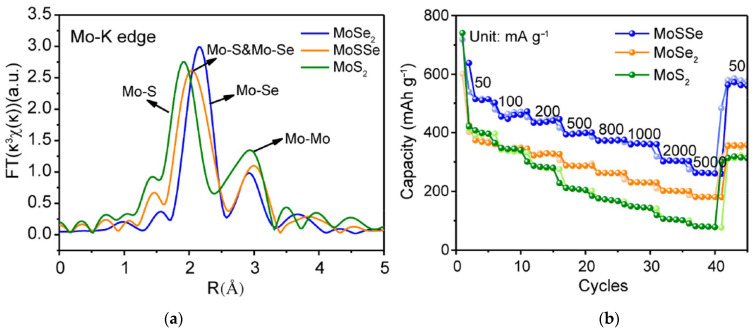
(**a**) Fourier transform magnitudes of EXAFS spectra measured for MoS_2_, MoSe_2_, and MoSSe; (**b**) cycling performance of MoS_2_, MoSe_2_, and MoSSe electrodes at various current densities in PIBs; taken with permission from [219]; Copyright 2019, American Chemical Society.

A number of works were devoted to the synthesis of Se-doped MoS_2_ anodes. Kang et al., made doped nanosheets using Sb powder in hydrothermal synthesis [217]. According to the HRTEM analysis, the sheets were separated by 0.96 nm, which ensured an increase in the reversible capacity of Sb-doped MoS_2_ at 2 A∙g^−1^ by 1.34 times as compared to the undoped electrode. Gao et al., prepared MoS_2−x_Se_x_ nanotubes coated with a thin carbon layer doped with nitrogen and phosphorus [218]. The best performance in PIBs was found for MoS_1.3_Se_0.7_, and an excess amount of selenium substituents caused a decrease in cell cycling stability. An ex-situ XRD study of the reaction products showed that the structure of MoS_1.3_Se_0.7_/N,P-carbon nanotubes is reversible during discharge/charge processes. Fan et al., synthesized spherical N-doped carbon-coated MoS_1.5_Se_0.5_ structures, which had a narrower band gap as compared to their undoped counterpart [216]. The MoS_1.5_Se_0.5_/NC anode showed long-term stability at a high current density of 5 Ag^−1^ over 500 cycles due to reversible formation of KMo_3_Se crystals. Fast reaction kinetics and high cycling stability was also demonstrated for the MoS_1.6_Se_0.4_/N-carbon anode [221]. The reversibility of the conversion reaction was associated with the inhibition of molybdenum agglomeration due to the presence of selenium. He et al., fabricated vacancy-enriched MoS_2−x_Se_x_ (x = 0.75, 0.5, 0.25) nanoplates by replacing selenium with sulfur at 700 °C [219]. The EXAFS study confirmed the substitution of S for Se at the atomic level (Figure 12a) The MoSSe alloy showed much better performance in PIB than MoS_2_ and MoSe_2_ (Figure 12b). 

Only two works were related to the synthesis of anodes for PIBs by replacing molybdenum in the MoS_2_ lattice. Kang et al., reported doping MoS_2_ with tellurium using Te powder in a hydrothermal process [220]. HRTEM analysis revealed that Te atoms occupy Mo positions with the formation of Te–S bonds, which increases the electrical conductivity of nanomaterial and causes the formation of 1T–1H in-plane heterojunctions. The nanomaterial demonstrated remarkable rate capability, delivering 395 mAh∙g^−1^ at 5 A∙g^−1^ with 88% capacity retention over 1000 cycles. A full cell with this anode and K_2_Fe[Fe(CN)_6_] cathode had a capacity of 61.7 mAh∙g^−1^ at a current density of 0.05 A∙g^−1^ after 50 cycles, demonstrating the practical application of Te-doped MoS_2_ in PIB. MoS_2_ co-doped with Fe and Co and inter-overlapped with N,O-doped graphene layers showed the best cycling performance at high current density (Table 5). This anode material was stable at a current density of 20 C and the fast transport of K^+^ ions was ascribed to a strong surface capacitive effect induced by the incorporation of magnetic atoms in the MoS_2_ lattice [198]. 

### 4.3. Supercapacitors

Unlike batteries, supercapacitors have a long lifespan and high power density, but insufficient energy density [222]. Therefore, efforts were focused on obtaining electrodes with good rate capability. The attractiveness of MoS_2_ as an electrode material for supercapacitors lies in the large interlayer space for the intercalation and diffusion of electrolyte ions and the variable oxidation states of Mo, which can participate in redox reactions [223]. Charge storage in MoS_2_ occurs due to the capacitance of the electric double-layer and the pseudocapacitive contribution [224,225]. Reviews on approaches to the synthesis of MoS_2_ nanomaterials, including their compositions with carbon derivatives, conducting polymers, metal sulfides, etc., for use in supercapacitors are given in [226,227]. The main disadvantage of MoS_2_ is its low electrical conductivity, which can be enhanced by combining the thermodynamically stable 2H phase and the metallic 1T phase [223]. Such a structural modification improves the performance of MoS_2_-based electrodes in supercapacitors; however, an analysis of the literature showed that the doping with heteroatoms is the best strategy for this [223,225]. 

Table 6 presents the highest specific capacitance values recorded for heteroatom-doped MoS_2_ electrodes using three-electrode systems, as well as power and energy densities obtained during two-electrode cell tests. All works mentioned an increase in the specific capacitance for doped MoS_2_ as compared to undoped MoS_2_ synthesized using the same synthetic approach. The reasons for this are an increase in the interlayer space due to the introduction of foreign elements, enhanced conductivity of the electrode material, the appearance of additional adsorption centers, and the contribution from redox-active substituents. An additional gain in the capacitance is achieved when the 1T phase is formed. The positive effect of this structural modification was observed in the case of N-doping [228,229], Mn-doping [230,231], Ni-doping [231,232], and Co-doping [231,233,234]. Simultaneous doping with two foreign elements is also beneficial. For example, the introduction of C, N [187] or Mn, Se [235] into the MoS_2_ lattice provided high values of specific capacitance, good rate capability, and capacitance retention during long-term cycling. 

An analysis of the literature showed that doping with Se, Ni, and Co allows to obtain a very high specific capacitance, approaching [239,246] or even exceeding [231] the theoretically expected value of 1500 F∙g^−1^ for MoS_2_. When designing an electrode material, the optimal ratio of Mo to heteroatom is often determined, and Table 6 provides data for the best achieved cases. The optimal content of Ni or Co in the MoS_2_ is 6–7 at% [231,232,247]. A comparative study of MoS_2_ nanomaterials doped with Ni, Fe, or Cu in a 6 M KOH electrolyte revealed the pseudocapacitive behavior of the Fe-doped electrode and predominance of electric double-layer capacitance for electrodes doped with Cu and Ni [244]. A study of kinetics of the electrochemical reactions occurring with Ni-doped MoS_2_ revealed a significant contribution of intercalation process, which is controlled by diffusion [243]. DFT calculations showed that strong interaction between Ni and S results in a distortion of MoS_2_ lattice and a redistribution of electron density. This interaction also causes a decrease in the number of adjacent layers in the nanomaterial, which increases the rate of insertion and extraction of electrolyte ions. 

**Figure 13:**
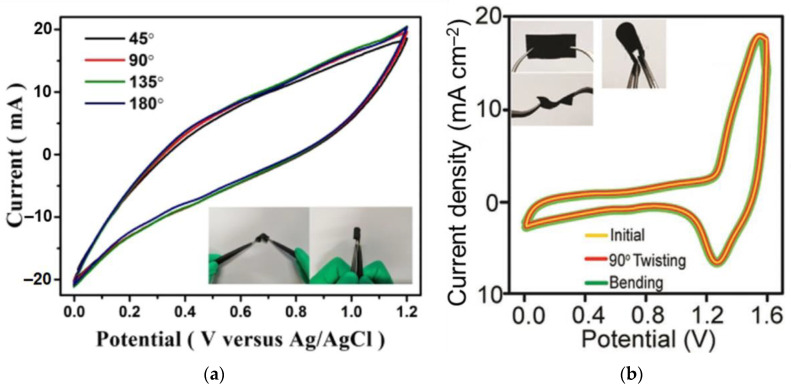
(**a**) CV curves measured under different bending angles of Pt-doped MoS_2_/carbon cloth electrode at 100 mV s^−1^; taken with permission from [248]; Copyright 2019, Elsevier B.V.; (**b**) CV curves of 1T-Mn_x_Mo_1−x_S_2−y_Se_y_/carbon cloth after bending and twisting of a supercapacitor; taken with permission from [235]; Copyright 2020, Wiley-VCH Verlag GmbH and Co; KGaA.

Heteroatom-doped MoS_2_ nanomaterials were used to make binder-free electrodes by depositing a sample on a nickel foam [241] or glassy carbon [245]. The procedure of forming a working electrode using electrodeposition looks promising, since it consist of a single step and a deposited film tightly bonded to the electrode surface, which ensures high operation stability of the cell. Flexible supercapacitors were fabricated from carbon cloth directly during hydrothermal synthesis [229,235,248] or by applying a nanomaterial dispersion [233]. Cyclic voltammetry (CV) curves of flexible solid-state supercapacitors showed no significant changes after bending and twisting tests (Figure 13), indicating that they can be used in wearable and foldable electrical devices.

The ideal energy devices should possess a high energy density at high power density. For heteroatom-doped MoS_2_, the best characteristics are obtained with the substitution of a half of sulfur atoms by selenium [239], and the incorporation of Mn [230,235] and Ni [231,232]. A symmetrical supercapacitor with Mn-doped MoS_2_ electrodes and a hybrid supercapacitor with a Ni-doped MoS_2_ positive electrode demonstrated practical applicability by lighting LED indicators [230,231].

## 5. Conclusions

Substitutional heteroatom doping is a common way to modify the structural, chemical, and electronic properties of a compound. Theory predicts that most elements of the periodic table can be introduced into the MoS_2_ lattice. The thermodynamically stable hexagonal form MoS_2_ is a semiconductor, and the introduction of an element from the same group as Mo or S, and, therefore, isovalent to the substituent reduces the band gap. Elements with a smaller (larger) number of the valence electrons induce impurity states near the valence (conductance) band. These states change the type of electrical conductivity of MoS_2_ and create reaction centers on its surface. Many elements that are theoretically suitable as substituents in MoS_2_ have been experimentally introduced into its structure. The rest of the elements present a challenge for experimentalists to find the necessary synthetic procedure. The electron density redistribution in MoS_2_ doped with heteroatoms makes the surface more active for adsorbates, which is an important factor, in particular, for sensor and electrochemical applications. In addition, the use of sources of foreign elements in the synthesis of MoS_2_ usually leads to the formation of few-layer nanomaterials with an increased interlayer space. This also increases the surface area of MoS_2_, which is also important for many applications. 

This review was devoted to experimental and theoretical studies of MoS_2_ nanomaterials in chemiresistive sensors and energy storage devices, such as LIBs, SIBs, PIBs, and supercapacitors, and, for the first time, focused exclusively on the effect of substitutional heteroatoms on these properties. Studies of thin layers of MoS_2_ showed their ability to detect some gases at the ppb level. In DFT calculations, sensitivity is defined as the strength of the sensor–analyte interaction, and calculations showed that this strength increases when MoS_2_ is doped with heteroatoms. Experimental and theoretical data agree that MoS_2_ sensors are most sensitive to nitrogen dioxide NO_2_, and substituents can provide selectivity to one or another gas. Increased interlayer spacing, better electrical conductivity, and surface polarization are key factors for the improved electrochemical performance typically seen with heteroatom-doped MoS_2_ electrodes. The best characteristics are obtained by doping with Se or magnetic metals Fe, Co, Ni. DFT calculations showed that the presence of these elements in the MoS_2_ lattice lowers the diffusion barrier for alkali metal ions, resulting in high rate capability of the battery. The rare examples currently available showed that co-doping with different elements is a fruitful approach to improve the performance of MoS_2_ in sensors and energy storage applications, and developments in this direction could be the next step towards creating novel amazing MoS_2_-based nanomaterials. 

To realize the full potential of heteroatom-doped MoS_2_ nanomaterials, several challenges need to be solved. The available studies showed that many foreign metals occupy sulfur vacancies, which are often realized in the MoS_2_ lattice during synthesis. Thus, additional efforts are required to find conditions for replacing molybdenum. It is extremely important to establish clear relationships between the property and the content and position of the substituent in the MoS_2_ lattice. At present, the composition of such nanomaterials is usually determined by EDS and XPS methods. The former method gives a significant error in the values and, in addition, the signals from some elements overlap. The latter method is surface-sensitive and can give distorted information for nanoparticles thicker than ~5 nm. Therefore, appropriate analytical chemical procedures should be developed and used. Direct visualization of foreign atoms in a crystal is only possible using the HRTEM and HAADF/STEM methods. These methods are local, and close contrasts of some elements make their identification difficult. Moreover, these methods are more suitable for thin films, like monolayers. The environment of the element under study, including the number of neighbors and the distance to them, is determined by the EXAFS method. However, in most cases, models are needed to determine the actual structure. Finding the right models can require extensive DFT calculations. 

The performance of heteroatom-doped MoS_2_ in batteries and supercapacitors is significantly improved by the addition of carbon component. This component also contributes to the accumulation processes, and this factor should be clearly defined for further successful design of nanomaterials. MoS_2_-based nanomaterials have great prospects as chemiresistive gas sensors and electrodes in electrochemical energy storage devices. Due to their excellent mechanical and chemical stability, they can be used in wearable electronics. It has been shown that supercapacitors with MoS_2_ electrodes retain their characteristics when bent and twisted. This property is also desirable for gas sensors that can be used in e-skin for on-site environmental monitoring. Substitutional doping with heteroatoms often improves the characteristics of MoS_2_, and this direction will undoubtedly be actively developed in the near future.

## Figures and Tables

**Table 1:** Comparison of heteroatom-doped MoS_2_ gas sensors with the best undoped MoS_2_ sensors.

Nanomaterial	Analyte	Detection Limit, Temperature	Reference
5% Zn-doped MoS_2_ nanoparticles	O_3_	17 ppb, 25 °C	[139]
	NO_2_	215 ppb, 25 °C	
Ni-doped MoS_2_ nanoparticles	SO_2_	250 ppb, room	[141]
7% Ni-doped MoS_2_ nanoparticles	NO_2_	311 ppb, room	[142]
MoSSe nanoparticles	NO	270 ppb, 25 °C	[143]
N-doped MoS_2_ nanosheets	NO_2_	125 ppb, 25 °C	[144]
Nb-doped MoS_2_ monolayer	NO_2_	5 ppm, 100 °C	[145]
6% Nb-doped MoS_2_ monolayer	N(CH_2_CH_3_)_3_	15 ppb, room	[146]
mechanically detached MoS_2_	NO	800 ppb, room	[110]
CVD MoS_2_ monolayer	NO_2_	1.4 ppb	[117]
CVD MoS_2_ monolayer	NO_2_ NH_3_	20 ppb, room 1 ppm, room	[116]
CVD MoS_2_ films	NH_3_	300 ppb, room	[114]
Edge-enriched MoS_2_	NO_2_ NH_3_	20 ppb, 100 °C 2 ppm, room	[122]
MoS_2_ nanowires	NO_2_	4.6 ppb, 60 °C	[123]
mechanically detached MoS_2_	N(CH_2_CH_3_)_3_	~1 ppm, 20 °C	[115]

**Table 2:** DFT calculated adsorption energy (E_ads_), charge transfer (ΔQ), and shortest distance (d) for molecules on the surface of heteroatom-doped MoS_2_.

Heteroatom	Gas Molecule	E_ads_ (eV)	ΔQ (e)	d (Å)	Correction	Reference
metal						
Fe	CO	−1.60	−0.27	1.86	D2	[149]
	NO	−2.84	−0.32	1.68		
	O_2_	−2.10	−0.70	-		
	NO_2_	−2.29	−0.66	2.11		
	NH_3_	−1.52	0.16	2.12		
Co	CO	−1.71	−0.19	1.80		
	NO	−2.95	−0.25	1.64		
	O_2_	−1.61	−0.54	-		
	NO_2_	−1.79	−0.62	2.04		
	NH_3_	−1.38	0.14	2.07		
Ni	CO	−1.69	−0.16	-		
	NO	−1.88	−0.22	-		
	O_2_	−0.98	−0.44	-		
	NO_2_	−1.32	−0.42	-		
	NH_3_	−1.33	0.15	-		
	C_2_H_4_O	−0.79	−0.19	2.13	D3	[150]
	CH_2_O	−1.08	0.02	1.85	D2	[151]
Cu	CO	−1.27	−0.02	-	D2	[149]
		−1.25	−0.08	1.86	D2	[152]
	NO	−1.5	−0.22	-	D2	[149]
		−1.44	−0.26	1.81	D2	[152]
	O_2_	−1.16	−0.46		D2	[149]
	NO_2_	−1.88	−0.64	-		
	NH_3_	−1.47	0.18	-		
Ag	CO	−0.79	−0.01	-		
		−0.81	0.02	2.09	D2	[153]
	NO	−0.98	−0.20	-	D2	[149]
		0.38	−0.13	2.18	D2	[154]
	O_2_	−0.62	−0.38	-	D2	[149]
	NO_2_	−1.49	−0.61	-		
		−1.85	−0.44	2.18	D2	[154]
		−2.83	−0.61	2.35	D2	[153]
	NH_3_	−1.12	0.14	-	D2	[149]
		−1.13	0.14	2.26	D2	[153]
	H_2_S	−0.85	0.19	2.52		
	SO_2_	−0.71	−0.35	2.44		
	CH_2_O	−0.73	0.01	2.27		
Au	CO	−1.06	−0.02	-	D2	[149]
	NO	−1.24	−0.20	-		
		−0.72	−0.11	2.16	D2	[154]
	O_2_	−0.74	−0.39	-	D2	[149]
	NO_2_	−1.70	−0.54	-		
		−1.60	−0.42	2.08	D2	[154]
	NH_3_	−1.17	0.23	-	D2	[149]
Au-Ag	NO	−0.55	−0.22	2.17	D2	[154]
	NO_2_	−2.60	−0.45	2.19		
Rh	CO	−1.49	−0.16	-	D2	[149]
	NO	−2.74	−0.20	-		
	O_2_	−1.29	−0.45	-		
	NO_2_	−1.64	−0.29	-		
	NH_3_	−1.20	0.19	-		
Pd	CO	−1.13	−0.06	-		
	NO	−1.05	−0.14	-		
	O_2_	−0.39	−0.17	-		
	NO_2_	−0.77	−0.34	-		
	NH_3_	−0.94	0.11	-		
	C_2_H_4_O	−0.53	0.13	2.31	D3	[150]
	CH_2_O	−0.57	0.09	2.13	D2	[151]
Pt	CO	−1.60	−0.08	-	D2	[149]
	NO	−1.36	−0.17	-		
	O_2_	−0.47	−0.26	-		
	NO_2_	−0.99	−0.31	-		
	NH_3_	−1.08	0.16	-		
	CH_2_O	−0.73	−0.03	2.1	D2	[151]
Ir	CO	−2.04	−0.16	-	D2	[149]
	NO	−3.36	−0.26	-		
	O_2_	−1.68	−0.55	-		
	NO_2_	−2.08	−0.40	-		
	NH_3_	−1.33	0.17	-		
Ti	C_2_H_4_O	−1.23	0.28	2.09	D3	[150]
	CH_2_O	−1.59	−0.37	1.86	D2	[151]
V	CO	−1.25	−0.24	-	D3	[155]
	NO_2_	−2.59	−0.66	-		
	H_2_O	−0.81	0.03	-		
	NH_3_	−1.30	0.1	-		
Nb	CO	−1.36	−0.33	-		
	NO_2_	−3.88	−0.69	-		
	H_2_O	−0.92	0.04	-		
	NH_3_	−1.24	0.1	-		
Ta	CO	−1.70	−0.35	-		
	NO_2_	−3.64	−0.72	-		
	H_2_O	−1.44	0.05	-		
	NH_3_	−1.71	0.1	-		
non-metal						
Si	C_2_H_4_O	−1.65	0.29	1.85	D3	[150]
	H_2_S	−0.68	−0.16	2.40	vdW	[156]
	SOF_2_	−3.63	0.83	1.64		
	NO_2_	−3.56	−0.30	2.37	D3	[157]
Ge	C_2_H_4_O	−0.24	0.02	3.23	D3	[150]
	N_2_	−0.11	0.01	3.88	D3	[158]
	O_2_	−0.22	0.12	2.52		
	H_2_S	−0.15	−0.01	3.84		
	NO_2_	−1.81	−0.17	2.82	D3	[157]
P	C_2_H_4_O	−0.13	0.03	2.85	D3	[150]
	N_2_	−0.06	0.01	3.21	D3	[158]
	O_2_	−0.16	0.06	3.01		
	H_2_S	−0.37	−0.20	2.84		
	NO_2_	−2.49	−0.66	2.42	D3	[157]
Cl	C_2_H_4_O	−0.21	0.02	3.26	D3	[150]
	N_2_	−0.09	0.01	3.41	D3	[158]
	O_2_	−0.01	0.30	2.52		
	H_2_S	−0.13	0.01	2.98		
	NO_2_	−1.53	−0.14	2.53	D3	[157]
Se	NO_2_	−0.23	−0.04	2.54		

**Table 3:** Performance of anodes from heteroatom-doped MoS_2_-based nanomaterials in lithium-ion batteries.

Nanomaterial	Initial Specific Capacity (Current Density)	Reversible Capacity (Current Density)	Reference
N-doped MoS_2_ nanoparticles	1130.8 mAh∙g^−1^ (0.2 C)	800 mAh∙g^−1^ (0.2 C) after 40 cycles	[182]
N-doped MoS_2_ nanoparticles	1186 mAh∙g^−1^ (0.1 C)	738 mAh∙g^−1^ (0.5 C) after 100 cycles	[183]
N-rGO/MoS_2_	726.9 mAh∙g^−1^ (1 C)	592.7 mAh∙g^−1^ (1 C) after 100 cycles	[184]
N-rGO/MoS_2_	1725.6 mAh∙g^−1^ (0.1 A∙g^−1^)	~630 mAh∙g^−1^ (0.5 A∙g^−1^) after 400 cycles	[185]
N, C-doped MoS_2_ nanoparticles	1280 mAh∙g^−1^ (0.1 A∙g^−1^)	400 mAh∙g^−1^ (0.1 A∙g^−1^) after 60 cycles	[187]
7.9% Sn-doped MoS_2_ flowers	1087 mAh∙g^−1^ (0.2 A∙g^−1^)	1087 mAh∙g^−1^ (0.2 A∙g^−1^) after 100 cycles	[189]
17.6% V-doped MoS_2_	814 mAh∙g^−1^ (1 A∙g^−1^)	350 mAh∙g^−1^ (1 A∙g^−1^) after 300 cycles	[190]
MoS_1.75_Se_0.25_	~1640 mAh∙g^−1^ (0.1 C)	500 mAh∙g^−1^ (5 C) after 350 cycles	[191]
meso-MoS_1.12_Se_0.88_/rGO	~1330 mAh∙g^−1^ (0.1 A∙g^−1^)	830 mAh∙g^−1^ (0.1 A∙g^−1^) after 150 cycles	[192]
P-doped MoS_2_/carbon cloth	2700 mAh∙g^−1^ (0.1 A∙g^−1^)	713 mAh∙g^−1^ (0.5 A∙g^−1^) after 500 cycles	[193]
Mn-doped MoS_2_/carbon cloth	~1280 mAh∙g^−1^ (0.1 A∙g^−1^)	~1130 mAh∙g^−1^ (0.1 A∙g^−1^) after 200 cycles	[194]
Fe-doped MoS_2_/rGO	1671 mAh∙g^−1^ (0.1 A∙g^−1^)	946 mAh∙g^−1^ (0.1 A∙g^−1^) after 100 cycles	[195]
Co-doped MoS_2_/rGO	1385.3 mAh∙g^−1^ (0.1 A∙g^−1^)	1223 mAh∙g^−1^ (0.1 A∙g^−1^) after 100 cycles	[196]
Co_1/3_Mo_2/3_S_2_/rGO	~1800 mAh∙g^−1^ (0.2 A∙g^−1^)	1200 mAh∙g^−1^ (0.2 A∙g^−1^) after 700 cycles	[197]
FeCo-doped MoS_2_/carbon	1874.4 mAh∙g^−1^ (0.1 C)	971.2 mAh∙g^−1^ (5 C) after 3000 cycles	[198]

**Table 4:** Performance of anodes from heteroatom-doped MoS_2_-based nanomaterials in sodium-ion batteries.

Nanomaterial	Initial Specific Capacity (Current Density)	Reversible Capacity (Current Density)	Reference
N-doped MoS_2_/C hollow nanostructures	972 mAh∙g^−1^ (0.1 A∙g^−1^)	128 mAh∙g^−1^ (2 A∙g^−1^) after 5000 cycles	[204]
N-doped MoS_2_ foam	1193 mAh∙g^−1^ (0.02 A∙g^−1^)	312.4 mAh∙g^−1^ (2 A∙g^−1^) after 100 cycles	[205]
N-doped MoS_2_/C spheres	745 mAh∙g^−1^ (0.1 C = 0.067 A∙g^−1^)	401 mAh∙g^−1^ (0.2 C) after 200 cycles	[206]
N-doped MoS_2_/C@SiOC	716.6 mAh∙g^−1^ (0.05 A∙g^−1^)	~680 mAh∙g^−1^ (0.1 A∙g^−1^) after 200 cycles	[207]
N-MoS_2_/N-carbon nanotubes	658 mAh∙g^−1^ (0.1 A∙g^−1^)	372.3 mAh∙g^−1^ (2 A∙g^−1^) after 100 cycles	[208]
N-rGO/MoS_2_	1100 mAh∙g^−1^ (0.1 A∙g^−1^)	542 mAh∙g^−1^ (0.2 A∙g^−1^) after 300 cycles	[203]
Sb-doped MoS_2_/N-carbon	~920 mAh∙g^−1^ (0.1 A∙g^−1^)	253 mAh∙g^−1^ (1 A∙g^−1^) after 2200 cycles	[209]
C@MoS_2−x_Te_x_@C	630.7 mAh∙g^−1^ (0.2 A∙g^−1^)	365.3 mAh∙g^−1^ (1 A∙g^−1^) after 300 cycles	[211]
Re-doped fullerene-like MoS_2_	~160 mAh∙g^−1^ (0.1 C = 0.02 A∙g^−1^)	74 mAh∙g^−1^ (20 C) after 30 cycles	[212]
Mn-doped MoS_2_ nanotubes	778 mAh∙g^−1^ (0.1 A∙g^−1^)	160 mAh∙g^−1^ (1 A∙g^−1^) after 1000 cycles	[213]
VMoS_2_ flowers	580.1 mAh∙g^−1^ (0.1 A∙g^−1^)	451.6 mAh∙g^−1^ (2 A∙g^−1^) after 800 cycles	[214]
orderly layered VMoS_2_	791.8 mAh∙g^−1^ (0.2 A∙g^−1^)	534.1 mAh∙g^−1^ (2 A∙g^−1^) after 190 cycles	[215]
Co_1/3_Mo_2/3_S_2_/rGO	~1050 mAh∙g^−1^ (0.1 A∙g^−1^)	529 mAh∙g^−1^ (1 A∙g^−1^) after 200 cycles	[197]
Ni-MoS_2_@porous carbon	930.1 mAh∙g^−1^ (0.1 A∙g^−1^)	337.5 mAh∙g^−1^ (1 A∙g^−1^) after 500 cycles	[210]
FeCo-doped MoS_2_/carbon	982.7 mAh∙g^−1^ (0.1 C)	473.3 mAh∙g^−1^ (5 C) after 3000 cycles	[198]

**Table 5:** Performance of anodes from heteroatom-doped MoS_2_-based nanomaterials in potassium-ion batteries.

Nanomaterial	Initial Specific Capacity (Current Density)	Reversible Capacity (Current Density)	Reference
1T Sb-doped MoS_2_/N-carbon	~700 mAh∙g^−1^ (0.1 A∙g^−1^)	343 mAh∙g^−1^ (0.1 A∙g^−1^) after 100 cycles	[209]
Se-doped MoS_2_ nanosheets	~320 mAh∙g^−1^ (0.05 A∙g^−1^)	140 mAh∙g^−1^ (1 A∙g^−1^) after 100 cycles	[217]
MoS_1.3_Se_0.7_/N,P-carbon nanotubes	686 mAh∙g^−1^ (0.2 A∙g^−1^)	237 mAh∙g^−1^ (0.5 A∙g^−1^) after 300 cycles	[218]
MoS_1.5_Se_0.5_/NC	~880 mAh∙g^−1^ (0.2 A∙g^−1^)	531.6 mAh∙g^−1^ (0.2 A∙g^−1^) after 1000 cycles	[216]
MoS_1.6_Se_0.4_/N-carbon	1671 mAh∙g^−1^ (0.1 A∙g^−1^)	143.7 mAh∙g^−1^ (2 A∙g^−1^) after 1500 cycles	[221]
Vacancy-rich MoSSe	701.6 mAh∙g^−1^ (0.1 A∙g^−1^)	~310 mAh∙g^−1^ (2 A∙g^−1^) after 1000 cycles	[219]
Te-doped MoS_2_	723.4 mAh∙g^−1^ (0.1 A∙g^−1^)	301 mAh∙g^−1^ (2 A∙g^−1^) after 1000 cycles	[220]
FeCo-doped MoS_2_/carbon	542.3 mAh∙g^−1^ (0.1 C)	209.8 mAh∙g^−1^ (5 C) after 3000 cycles	[198]

**Table 6:** Supercapacitor performance of heteroatom-doped MoS_2_-based nanomaterial electrodes.

Nanomaterial	Electrolyte	Specific Capacitance	Energy Density	Power Density	Reference
N-doped MoS_2_	1 M H_2_SO_4_	74.4 F∙g^−1^ at 2 A∙g^−1^	–	–	[228]
1T/2H N-doped MoS_2_ on carbon cloth	0.5 M H_2_SO_4_	410 F∙g^−1^ at 1 A∙g^−1^	–	–	[229]
N,C-doped MoS_2_ nanoflakes	6 M KOH	1400 F∙g^−1^ at 1 A∙g^−1^	45 Wh∙kg^−1^	912 W∙kg^−1^	[187]
O-doped MoS_2_ microspheres	1 M KCl	744.2 F∙g^−1^ at 1 A∙g^−1^	–	–	[236]
1T/2H O-doped MoS_2_/graphite foil	1 M Na_2_SO_4_	280 F∙g^−1^ at 1 A∙g^−1^	39.7 Wh∙kg^−1^	450 W∙kg^−1^	[237]
1T-MoSSe	6 M KOH	36 F∙g^−1^ at 0.5 A∙g^−1^	~12.1 Wh∙kg^−1^	~842.5 W∙kg^−1^	[238]
MoSSe	6 M KOH	1020 F∙g^−1^ at 10 A∙g^−1^	51 Wh∙kg^−1^	6000 W∙kg^−1^	[239]
Mo_0.91_W_0.09_S_2_/C_4_	1 M Na_2_SO_4_	432.7 F∙g^−1^ at 1 A∙g^−1^	–	–	[240]
Mn-doped MoS_2_ nanoflowers	0.5 M Na_2_SO_4_	351 F∙g^−1^ at 1 A∙g^−1^	48.9 Wh∙kg^−1^	5000 W∙kg^−1^	[230]
binder-free Mn-doped MoS_2_	1 M KOH	70.37 F∙g^−1^ at 1 A∙g^−1^	3.14 Wh∙kg^−1^	4346.35 W∙kg^−1^	[241]
1T Mn-doped MoS_2_	1 M KOH	980 F∙g^−1^ at 1 A∙g^−1^	–	–	[231]
1T Mn_x_Mo_1−x_S_2−y_Se_y_/carbon cloth	3 M KOH	~288 mAh∙g^−1^ at 1 mA cm^−2^	~69 Wh∙kg^−1^	985 W∙kg^−1^	[235]
Ni-doped MoS_2_	1 M Na_2_SO_4_	291 F∙g^−1^ at 0.5 A∙g^−1^	–	–	[242]
Ni-doped MoS_2_ microspheres	1 M Na_2_SO_4_	425 F∙g^−1^ at 5 mV∙s^−1^	9 Wh∙kg^−1^	0.5 W∙kg^−1^	[243]
6% Ni-doped MoS_2_	3 M KOH	528.7 F∙g^−1^ at 1 A∙g^−1^	140.9 Wh∙kg^−1^	11,520 W∙kg^−1^	[232]
1T Ni-doped MoS_2_	1 M KOH	2461.2 F∙g^−1^ at 1 A∙g^−1^	65.96 Wh∙kg^−1^	700 W∙kg^−1^	[231]
Ni-doped MoS_2_	6 M KOH	285 F∙g^−1^ at 1 A∙g^−1^	4.83 Wh∙kg^−1^	2660 W∙kg^−1^	[244]
Fe-doped MoS_2_	6 M KOH	211 F∙g^−1^ at 1 A∙g^−1^	4.08 Wh∙kg^−1^	6000 W∙kg^−1^	[244]
Cu-doped MoS_2_	6 M KOH	353 F∙g^−1^ at 1 A∙g^−1^	5.58 Wh∙kg^−1^	6000 W∙kg^−1^	[244]
Cu-doped MoS_2_ film	1 M Na_2_SO_4_	502 F∙g^−1^ at 1 A∙g^−1^	–	–	[245]
Co-doped MoS_2_	2 M KOH	510 F∙g^−1^ at 1 A∙g^−1^	–	–	[234]
Mo_0.7_Co_0.3_S_2_/g-C_3_N_4_	5 M KOH	1063.22 F∙g^−1^ at 0.5 A∙g^−1^	–	–	[246]
Mo_0.7_Co_0.3_S_2_		822.1 F∙g^−1^ at 0.5 A∙g^−1^	–	–	[247]
Co-doped MoS_2_ nanoflowers	1 M KOH	* 86 F∙g^−1^ at 1 A∙g^−1^	4.3 Wh∙kg^−1^	600 W∙kg^−1^	[233]
1T Co-doped MoS_2_	1 M KOH	1270.8 F∙g^−1^ at 1 A∙g^−1^	–	–	[231]
flexible Pt-doped MoS_2_	1 M Na_2_SO_4_	250 F∙g^−1^ at 0.5 A∙g^−1^			[248]

* obtained for two-electrode system.

## Data Availability

Data is contained within the article.
